# Ligand-Specific Remodeling of the *Anopheles gambiae* RDL GABA Receptor by the A296S Mutation Differentiates Recognition of Fipronil and Fluralaner

**DOI:** 10.3390/biology15212011

**Published:** 2026-11-05

**Authors:** Rasha K. Al-Akeel, Ibrahim M. Ibrahim, Abeer M. Alkhaibari, Amr Mohamed, Wei Zhang, Makio Takeda, Ahmed A. Ezat

**Affiliations:** 1 Department of Zoology, Faculty of Science, King Saud University, Riyadh 11451, Saudi Arabia; ralogial@ksu.edu.sa; 2 Biophysics Department, Faculty of Science, Cairo University, Giza 12613, Egypt; ibrahim_mohamed@cu.edu.eg; 3 Department of Biology, Faculty of Science, University of Tabuk, Tabuk 71491, Saudi Arabia; aalkhaibari@ut.edu.sa; 4 Department of Entomology, Faculty of Science, Cairo University, Giza 12613, Egypt; 5 State Key Laboratory of Green Pesticides, Guizhou University, Huaxi District, Guiyang 550025, China; wzhang9@gzu.edu.cn; 6 Graduate School of Agricultural Science, Kobe University, 1-1 Rokkodai-cho, Nada-ku, Kobe 657-8501, Japan; mtakeda@kobe-u.ac.jp

**Keywords:** RDL receptor, molecular dynamics simulation, MM/PBSA, ligand-gated ion channel, insecticide resistance, cross-resistance

## Abstract

The γ-aminobutyric acid (GABA)-gated chloride channel Resistance to Dieldrin (RDL) receptor is a major molecular target of insecticides, yet the structural basis by which resistance-associated mutations differentially influence chemically distinct ligands remains incompletely understood. Here, we investigated the effects of the classical A296S substitution on the interactions of the *Anopheles gambiae* RDL receptor with the phenylpyrazole fipronil and the isoxazoline fluralaner using homology modeling, molecular docking, 200 ns molecular dynamics simulations (apo receptors and three independent replicas of each ligand-bound complex), and MM/PBSA free-energy calculations that include an interaction-entropy (IE) term. Apo-state molecular dynamics simulations showed that A296S preserved the overall structural stability of the receptor while locally modifying the environment of the pore-lining M2 transmembrane helix. Although docking scored fluralaner more favorably than fipronil in both receptor variants, the replicated molecular dynamics simulations revealed ligand- and genotype-dependent behavior. In the wild-type receptor, fluralaner remained stably bound and had a favorable MM/PBSA free-energy estimate (−18.3 ± 4.2 kcal/mol), whereas the ligand RMSD of fipronil increased progressively and its estimate was close to zero (+1.2 ± 9.5 kcal/mol). In the A296S model, the fipronil estimate was similar (+1.6 ± 4.3 kcal/mol), but the fluralaner estimate became unfavorable (+10.0 ± 1.8 kcal/mol), and Ser296 itself contributed slightly unfavorably to both ligands. Together, these findings indicate that A296S does not act as a universal determinant of ligand recognition but is associated with ligand-specific changes in the modeled interaction landscape, with the larger predicted effect on fluralaner. These predictions derive from a homology model, were not tested by electrophysiology or binding assays, and do not measure functional resistance. They may nonetheless help inform the interpretation of cross-resistance patterns and structure-guided efforts targeting GABA receptor-based insecticides.

## Simple Summary

Malaria mosquitoes can develop insecticide resistance through small changes in the proteins these chemicals target. One such change, A296S, occurs in a receptor that mediates inhibitory nerve signaling and is linked to resistance against certain insecticide classes, though why it affects different insecticides unevenly has remained unclear. Using structural modeling and molecular simulations, we compared how two insecticides, fipronil and fluralaner, interact with this receptor in its normal and mutated forms. In repeated simulations, A296S was predicted to alter the local chemical environment of the binding site and to weaken the predicted binding of fluralaner, whereas the predicted binding of fipronil was weak in both receptor forms and did not change detectably. These findings offer molecular insight into how a single resistance-associated mutation can affect chemically distinct insecticides unevenly. These are computational predictions that have not been tested in laboratory assays, but they may help inform future efforts to design compounds less affected by this resistance mechanism.

## 1. Introduction

*Anopheles gambiae* (Insecta: Diptera: Culicidae) is a principal mosquito vector of *Plasmodium falciparum* malaria in sub-Saharan Africa, where malaria remains a major public-health burden. In 2024, the World Health Organization (WHO) African Region accounted for an estimated 94% of malaria cases and 95% of malaria deaths recorded globally, with approximately 75% of malaria deaths in the region occurring among children under five years of age [1]. This continued burden underscores the reliance on insecticide-based vector control and the corresponding threat posed by resistance at insecticide molecular targets in the mosquito nervous system [1].

Gamma-aminobutyric acid (GABA)-gated chloride channels mediate inhibitory neurotransmission in the insect nervous system, and the Resistance to Dieldrin (RDL) receptor is their best-characterized member, serving as the principal postsynaptic GABA receptor in the insect central nervous system [2,3]. Binding of GABA opens the channel and permits chloride influx, thereby restraining neuronal excitability. Disruption of this inhibitory signaling causes hyperexcitation, convulsions, and death, making RDL a major molecular target of several classes of insecticides. These include the phenylpyrazole (fiprole) class, represented by fipronil, which acts as a non-competitive antagonist (NCA) of GABA-gated chloride channels and is classified by the Insecticide Resistance Action Committee (IRAC) within Group 2B (phenylpyrazoles/fiproles), under the broader Group 2 classification of GABA-gated chloride channel blockers, which also includes the earlier cyclodiene organochlorines (Group 2A) [4,5]. RDL is also targeted by the newer isoxazoline class, represented by fluralaner and classified within IRAC Group 30 as GABA-gated chloride channel allosteric modulators [4,5].

Structural and mutational studies have substantially refined the classical view that these NCAs act primarily through pore occlusion. The M2 transmembrane helix contains key determinants of ligand binding, with the conserved 2′ alanine residue, corresponding to A296 in representative dipteran numbering and A302 in *Drosophila melanogaster*, playing an important role in receptor sensitivity [3,6]. More recently, cryo-electron microscopy structures of insect RDL receptors, including a near-atomic-resolution honeybee receptor, have provided detailed insight into how the transmembrane domain accommodates both orthosteric ligands and allosteric modulators [3].

Isoxazolines such as fluralaner occupy an overlapping but functionally distinct site, which helps explain their retained efficacy against many resistant RDL alleles [7]. Rather than occupying the canonical pore-blocking pocket associated with cyclodienes and phenylpyrazoles, they bind at a transmembrane inter-subunit cavity centered at the M1–M3 interface, with potential contributions from residues within the deeper M2 segment [3,7]. This binding architecture is thought to contribute to their favorable selectivity for arthropod over vertebrate GABA receptors and their generally limited cross-resistance with older NCAs [7]. Nevertheless, these pharmacological sites are not completely independent. Structural and functional evidence indicates that residues surrounding the 2′ position contribute to both binding environments, suggesting that a single substitution could differentially influence ligand recognition across multiple insecticide classes [6,7].

The A296S substitution provides a well-characterized example of this phenomenon. First identified in cyclodiene-resistant *Drosophila* strains [8], it has subsequently been reported in diverse mosquito vector species [9,10,11]. Its effect on cyclodiene resistance is strong and well established. In contrast, its influence on fipronil susceptibility is considerably more variable, with studies reporting low-to-moderate cross-resistance, limited effects, or dependence on additional RDL substitutions and genetic background [6,12,13]. Evidence regarding isoxazolines remains comparatively limited, although recent studies suggest that A296S can influence fluralaner sensitivity without fully resolving the structural basis of this effect [7].

These observations highlight an important unresolved mechanistic question: how can a single conservative substitution at the 2′ position produce markedly different effects on ligands that interact with partially overlapping but non-identical binding sites? Phenotypic resistance assays can describe these divergent responses but cannot explain their molecular basis, which requires analysis of receptor–ligand interactions at atomic resolution.

Fipronil and fluralaner provide a deliberately contrasting test case for this question. Fipronil belongs to the phenylpyrazole class that succeeded cyclodienes as the dominant RDL-targeting chemistry [4], and shows the variable A296S-associated cross-resistance described above, whereas fluralaner is a structurally and pharmacologically distinct chemistry with emerging relevance as a candidate endectocide for *Anopheles* vector control [14,15]. Comparing their interactions with the same mutation therefore offers a computational means of exploring whether a resistance allele selected against older chemistry compromises the utility of a mechanistically distinct candidate insecticide whose response to this locus remains largely uncharacterized. Molecular dynamics (MD) simulations combined with Molecular Mechanics/Poisson–Boltzmann Surface Area (MM/PBSA) free-energy calculations provide a suitable framework for this comparison, enabling conformational sampling of receptor–ligand complexes and estimation of residue-level energetic contributions to binding [16].

Recent integrated in silico workflows in neuromuscular pharmacology have combined molecular docking and molecular dynamics simulations with complementary biophysical validation to characterize ligand-target interactions, illustrating the broader applicability of multistage computational approaches in pharmacological research [17].

Here, we investigated how the A296S mutation modulates the interaction landscape of the RDL receptor with fipronil and fluralaner using molecular docking, MD simulations, MM/PBSA free-energy calculations, and residue-level energetic decomposition. In the resulting models, A296S had little effect on the free-energy estimate for fipronil but was associated with markedly less favorable energetics for fluralaner, and the per-residue contributions changed in a ligand-specific manner. These findings offer atomistic insight into the ligand-specific effects of the A296S mutation and into how alterations within the transmembrane domain contribute to the molecular basis of target-site insecticide resistance.

## 2. Materials and Methods

### 2.1. Structural Modeling of the Anopheles gambiae RDL Receptor

No experimentally resolved structure of the *Anopheles gambiae* RDL receptor (UniProt accession: A0A1I9VT68) is currently available. A homology-based monomeric model was therefore generated as the structural framework for subsequent membrane simulations and ligand interaction analyses, using the Phyre2.2 webserver (accessed on 2 January 2026) [18]. The model was built on the 7QN8 template, chain D, with 48% sequence identity over 326 aligned residues. PDB 7QN8 is a cryo-EM structure of the human β3δ GABA(A) receptor determined in the presence of histamine and nanobody Nb25 [19]; chain D is a β3 subunit. The template is therefore a vertebrate heteromeric receptor and not an insect RDL structure, and its conformational state is inherited by the model. This monomeric model was used to construct the full pentameric assembly, consistent with the native architecture of pentameric ligand-gated ion channels (pLGICs), via the GalaxyHomomer webserver [20]. To improve local geometry and Ramachandran plot statistics, the resulting pentamers were subjected to structural refinement using the GalaxyRefine webserver, and the highest-quality model, based on refinement scoring metrics, was retained for all downstream analyses [21,22]. For GalaxyHomomer, the monomer PDB file was uploaded and the number of subunits was selected; for GalaxyRefine, the pentamer PDB file was uploaded and refined directly. As supplementary structural validation, the quality of the Phyre2.2 monomer, the refined homopentamer, a full-length AlphaFold2 monomer retrieved from the AlphaFold Protein Structure Database (entry AF-A0A1I9VT68-F1) [23,24], and a second monomer built on the honeybee RDL cryo-EM structure (PDB 9SIO [3]) was assessed with the structure assessment server [25], and the models were compared with TM-align [26] (Supplementary Table S1 and Figures S6–S8). The Phyre2.2 monomer was superposed on the AlphaFold monomer with an RMSD of 1.38 Å and a TM-score of 0.969, and on the honeybee-template monomer with an RMSD of 1.65 Å and a TM-score of 0.938. The refined homopentamer was superposed on the honeybee RDL structure with an RMSD of 2.24 Å, a TM-score of 0.94, and a global QS-score of 0.71. The pentamer comparison also gave an lDDT of 0.67, a local-distance measure. Together, these values indicate agreement of the overall fold. The honeybee structure 9SIO is an apo structure, whereas the 7QN8 template was determined with histamine bound, so part of the pentamer deviation may reflect a difference in conformational state. No M2-specific or residue 296-specific deviation was calculated, and the AlphaFold PAE matrix, which exists only for the AlphaFold prediction and can be viewed on its database entry, was not analyzed. The comparison therefore does not establish equivalence of the M1–M3 binding pocket and does not validate the docking poses or free-energy estimates. The receptor model has not been validated experimentally for *An*. *gambiae* RDL, and the results remain dependent on the conformational state of the template and on model construction.

### 2.2. Generation of the A296S Mutant Receptor

The A296S substitution, corresponding to the 2′ position of the M2 transmembrane helix, was introduced into the refined pentameric model using PyMOL, Version 3.1. Three-dimensional structures of the two insecticide ligands examined in this study, fipronil (PubChem CID: 3352) and fluralaner (PubChem CID: 25144319), were retrieved from the PubChem database for subsequent docking and simulation analyses.

### 2.3. Membrane System Construction and Molecular Dynamics Simulations

To sample the conformational landscape of the wild-type (WT) and A296S mutant receptors in their apo (unliganded) states, and subsequently to evaluate the stability of docked ligand poses, molecular dynamics (MD) simulations were performed using GROMACS 2023 [27,28]. Because RDL is an integral membrane protein whose function depends on its native lipid environment, both systems were embedded in a fully hydrated 1-palmitoyl-2-oleoyl-*sn*-glycero-3-phosphocholine (POPC) lipid bilayer. The spatial orientation of the protein relative to the membrane plane was determined using the Orientation of Proteins in Membranes (OPM) approach implemented via the PPM tool on the CHARMM-GUI webserver [29]. Explicit membrane embedding helps preserve the transmembrane context relevant to receptor conformational behavior, thermodynamic equilibria, and ligand-receptor dynamics during molecular dynamics simulations [30,31]. The complete membrane-protein assemblies were built using the CHARMM-GUI Membrane Builder module [32,33,34,35,36,37].

The WT simulation cell measured 12.5 × 12.5 × 20.8 nm along the X, Y, and Z axes, respectively, and contained 192 POPC molecules in the upper leaflet and 182 in the lower leaflet. The corresponding mutant system measured 12.5 × 12.5 × 20.7 nm, with 194 and 183 POPC molecules in the upper and lower leaflets, respectively. The ligand-bound WT-fluralaner, WT-fipronil, A296S-fluralaner, and A296S-fipronil systems contained 299,661, 300,066, 295,970, and 299,220 atoms in total, including 74,148, 74,290, 72,782, and 73,873 water molecules and 597, 598, 595, and 595 ions, respectively. System parameterization employed the AMBER FF19SB force field for the protein [38], Lipid21 for the POPC bilayer [39], GAFF2 for the small-molecule ligands [40], and the TIP3P model for explicit solvation. The overall simulation workflow comprised energy minimization, a six-stage equilibration protocol, and an unrestrained production phase. The present workflow likewise integrates receptor modeling, molecular docking, molecular dynamics simulations, and end-state free-energy analysis, consistent with recent multistage computational approaches used in pharmacological target characterization [17].

Energy minimization was carried out using the steepest descent algorithm until either 1,000,000 steps were completed or the maximum force fell below 10 kJ/(mol·nm^2^), whichever occurred first. Position restraints of 4000 kJ/(mol·nm^2^) and 2000 kJ/(mol·nm^2^) were applied to the protein backbone and side chains, respectively, together with a restraint of 1000 kJ/(mol·nm^2^) on lipid phosphorus atoms.

Equilibration proceeded through six sequential stages designed to progressively relax restraints while allowing stable adaptation of the protein, ligand, and lipid components. Phase 1 (2 ns, 1 fs time step, canonical NVT ensemble) maintained the temperature at 310 K using the V-rescale thermostat [41], with all hydrogen-containing covalent bonds constrained via the LINCS algorithm [42]; restraints matched those used during minimization. Phase 2 retained these parameters while reducing restraints to 2000, 1000, and 400 kJ/(mol·nm^2^) for the backbone, side chains, and lipid phosphorus atoms, respectively. Phase 3 transitioned the system to an isothermal-isobaric (NPT) ensemble, coupling the V-rescale thermostat (310 K) with a semi-isotropic C-rescale barostat [43] referenced to 1.0 atm, over 2 ns, with restraints further reduced to 1000, 500, and 400 kJ/(mol·nm^2^). Phase 4 continued under identical NPT conditions with restraints of 500, 200, and 200 kJ/(mol·nm^2^). Phase 5 further reduced restraints to 200, 50, and 40 kJ/(mol·nm^2^). In the final stage, Phase 6, the backbone restraint was reduced to 50 kJ/(mol·nm^2^) while restraints on side chains and lipid phosphorus atoms were fully released (0 kJ/(mol·nm^2^)), under the same NPT parameters. Following equilibration, an unrestrained production simulation was performed for 200 ns in the NPT ensemble using a 2 fs time step, with all position restraints removed to permit conformational sampling of the complete system. Temperature and pressure coupling time constants were 1.0 ps and 5.0 ps, respectively, and a cutoff of 0.9 nm was used for PME electrostatics and van der Waals interactions. In the ligand-bound systems, position restraints on the ligand during equilibration used the same force constant as that applied to the protein backbone at each stage; no restraints were applied during production.

### 2.4. Trajectory Analysis and Conformational Clustering

Following the 200 ns production simulations, conformational clustering of the protein backbone atoms from both the WT and mutant apo trajectories was performed using the TTClust Python library (version 4.10.4) under Python 3.12 [44] with Ward linkage applied to the backbone atoms, and the optimal cluster number was determined automatically via the integrated elbow method. A representative structure was extracted from each cluster to serve as the receptor conformation for subsequent molecular docking.

### 2.5. Molecular Docking Analysis

Ligand structures and receptor cluster representatives were converted to PDBQT format using Open Babel(version 3.1.1) [45], including assignment of atom types and addition of polar hydrogens. Docking was performed with the receptor held rigid while full conformational flexibility was permitted for both ligands. The binding pocket was defined using residues P292, A293, R294, V295, A296, L297, G298, V299, T300, T301, V302, L303, and T304 across all five chains, consistent with previously established RDL ligand-binding site boundaries [46]. The search space was centered at X = 56, Y = 59, Z = 111 Å within a 30 Å cubic grid box (Figure S5). Docking was performed using AutoDock Vina v1.2.7 with an exhaustiveness of 16 [47,48]. Docking was repeated for each representative conformation from the apo trajectories (six for the A296S receptor and five for the WT receptor), so receptor flexibility was sampled only through this ensemble of rigid structures. The grid box was intended to cover the M1–M3 inter-subunit cavity (Figure S5), which indicates coverage of the search space but does not establish that any docked pose is correct. No quantitative analysis of pose consistency across representative conformations was performed. For each ligand-receptor combination, the highest-affinity pose was selected for subsequent molecular dynamics simulation to assess binding stability under physiological conditions. Poses were selected by docking score alone, and the same site placement was not imposed on the WT and A296S complexes, because the receptor conformations differ and the hydroxyl group of Ser296 may constrict the search space in the mutant.

### 2.6. Ligand-Bound Molecular Dynamics Simulations

The top-ranked docking pose for each ligand-receptor combination (WT and A296S mutant, each with fipronil and fluralaner) was subjected to a 200 ns production MD simulation, following the same system preparation, force field parameterization, and simulation protocol described above for the apo systems. Each ligand-bound system was simulated in three independent 200 ns replicas built and equilibrated once from the same docked pose, and the production runs were started with different initial velocities. The apo WT and A296S systems were each simulated once and were not replicated, because this would have required repeating the clustering and docking steps.

### 2.7. MM/PBSA Binding Free-Energy Calculations

To quantify the total binding affinity (ΔGtotal) of the wild-type (WT) and mutant protein-membrane complexes with both ligands, Molecular Mechanics/Poisson–Boltzmann Surface Area (MM/PBSA) calculations were performed utilizing the gmx_MMPBSA tool, Version 1.7.0 [49,50]. In this scheme, the polar solvation contribution was obtained by numerically solving the linear Poisson–Boltzmann (PB) equation within an implicit-membrane representation, in which the membrane was treated as a slab-like continuum region interposed between two high-dielectric aqueous phases. The solute interior and solvent dielectric constants were set to 1.0 and 78.5, respectively, and the calculations were executed at the physiological temperature of 310 K and ionic strength of 0.150 M NaCl. The dielectric interface between the solute and the solvent was constructed with the classical geometric approach (ipb = 1), and the total electrostatic energy was evaluated using the particle–particle–particle–mesh (P3M) procedure of Lu and Luo [51], employing an atom-based cutoff of 7.0 Å for the short-range finite-difference interactions (cutfd) and a 99.0 Å cutoff for the van der Waals interactions (cutnb). The linear PB equation was discretized on a finite-difference grid whose extent was scaled to the dimensions of the solute by a fill ratio of 1.25 and solved using a geometric multigrid algorithm (solvopt = 2) under periodic boundary conditions (bcopt = 10) for a maximum of 1000 iterations (linit = 1000); no electrostatic focusing was applied (nfocus = 1).

The bilayer was represented implicitly using the heterogeneous dielectric membrane model, in which the membrane is described as a slab-like region whose dielectric constant varies continuously with depth from a value of 1 at the membrane center to 80 at the membrane surface, according to a PCHIP depth profile [52]; the nominal membrane dielectric constant was set to 7.0. The position of the membrane center and its thickness were determined automatically from the z-coordinates of the phosphorus atoms of the lipid head groups over the selected trajectory frames, with the membrane normal oriented along the *z*-axis, and the transmembrane aqueous pore was identified automatically using the search algorithm [49]. The non-polar solvation free energy was computed using the two-term decomposition scheme (inp = 2), expressed as the sum of a cavity term, linearly proportional to the solvent-accessible surface area (SASA), and a dispersion term evaluated by a surface-based integration method [53]; the molecular solvent-excluded surface was employed for the dielectric mapping and SASA calculations (sasopt = 0).

The entropic term (−TΔS) was estimated using the interaction entropy (IE) method [49,54]. It is not a normal-mode entropy.

The total binding free energy was derived from an ensemble average of 100 structural configurations extracted from the last 100 ns with an interval of 10 frames (one snapshot every one ns) for each of the three replicas, and results are reported as the mean ± standard deviation across replicas, according to the following equation:

(1) ∆Gtotal = ⟨Gcomplex − (Greceptor + Gligand)⟩

In this expression, the angle brackets represent the ensemble averages of the free energies for the complex, receptor, and ligand as sampled from the molecular dynamics (MD) trajectory. The underlying energetic components, including van der Waals forces, electrostatic interactions, and solvation energies, were determined through the following equations (Equations (2)–(6)):

(2) ∆Gtotal = ∆H − T∆S

(3) ∆H = ∆Egas + ∆Esol

(4) ∆Egas = ∆Eele + ∆EvdW

(5) ∆Esolv = EPB + ESA

(6) ESA = Ecavity + Edispersion

where ΔH represents the enthalpy, which is derived from the gas-phase energy (Egas) and the solvation-free energy (Esol). The term −TΔS accounts for the entropic contribution to the binding free energy. The gas-phase energy (Egas) comprises the electrostatic (Eele) and van der Waals (EvdW) energy terms. The solvation-free energy (Esol) is calculated as the sum of the polar solvation energy (EPB), obtained from the linear PB equation, and the nonpolar solvation energy (ESA), which combines the cavity and dispersion contributions. The free energy reported as ΔG bind (IE) is the enthalpic term (ΔTotal) plus the IE term, whereas ΔTotal alone is an enthalpic estimate. Specifically, ΔEnpolar denotes the cavity term and ΔEdisper the dispersion term of ESA, ΔGgas is the sum of ΔEeel and ΔEvdW, and ΔGsolv is the sum of ΔEPB and ESA.

### 2.8. Per-Residue Energy Decomposition Analysis

To identify the specific residues driving the differential binding energetics observed between complexes, a per-residue free-energy decomposition was performed for all amino acids located within 0.5 nm of the bound ligand, using the same MM/PBSA framework described above.

### 2.9. HOLE Analysis for the Protein–Ligand Complexes

MDAnalysis version 2.9 was used to measure the pore radius as a function of the z position along the trajectories of the ligand-bound complexes with the HOLE program [55,56,57] as implemented in MDAnalysis; the ligand was present in the calculation. Profiles are shown as the mean ± SD across the three replicas of each system. They describe the geometry of the modeled complexes only. No ion-permeation, conductance, or gating simulations were performed, so the profiles do not establish channel conductance or functional pore occlusion [55,56,57].

## 3. Results

### 3.1. Structural Modeling Reveals Preservation of Overall RDL Architecture Following A296S Substitution

**Figure 1:**
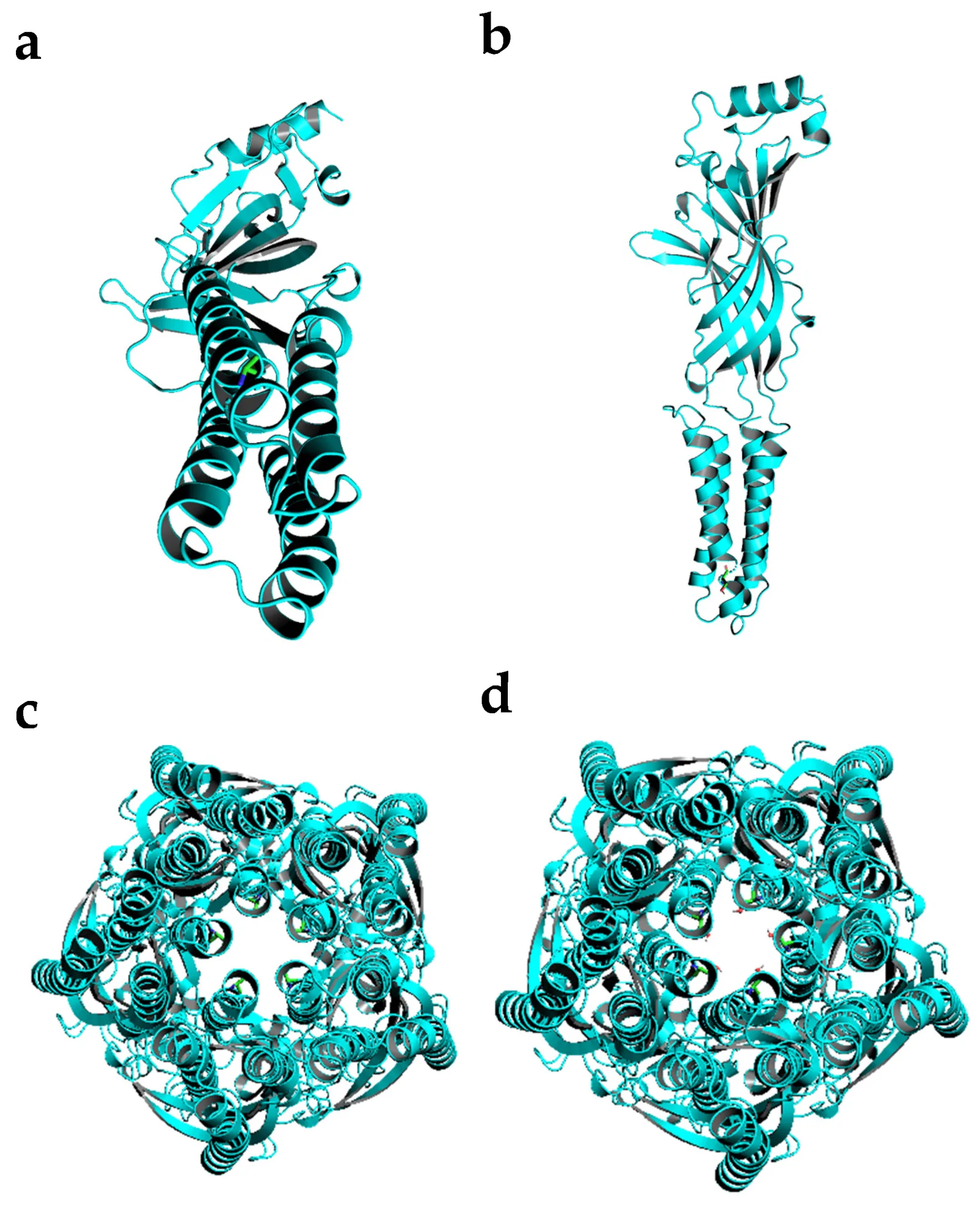
Homology-based structural models of the *Anopheles gambiae* RDL receptor. (**a**,**b**) Monomeric wild-type (WT) and A296S mutant subunits, respectively, showing the canonical RDL fold within the pentameric ligand-gated ion channel (pLGIC) family, comprising the N-terminal extracellular beta-sandwich domain and the four-helix transmembrane bundle; the C-terminal intracellular domain, largely disordered and unresolved in available template structures, is not represented. (**c**,**d**) WT and A296S mutant homopentamers, respectively, viewed down the central ion-conduction axis, illustrating the five-fold symmetric channel architecture characteristic of RDL GABA receptors. In all panels, residue 296 (Ala in WT, Ser in the mutant) is highlighted at the pore-facing 2′ position of the M2 helix to indicate the site of substitution.

In the absence of an experimentally resolved *An. gambiae* RDL structure, homology-based modeling was used to generate both WT and A296S mutant receptor models. The resulting monomer exhibited the canonical fold of the pLGIC family, comprising an N-terminal extracellular domain, four transmembrane helices, and a largely disordered C-terminal intracellular domain. This intracellular segment, which is not resolved in available template structures and lies distal to the insecticide-binding site, was excluded from the model (Figure 1a,b). The corresponding homopentameric assembly recapitulated the expected ion channel architecture of RDL GABA receptors (Figure 1c,d). Introduction of the A296S substitution did not disrupt this overall architecture; however, the additional hydroxyl group contributed by the substituted serine side chain was oriented toward the channel lumen, consistent with a modest narrowing of the predicted pore diameter relative to the WT structure. The overall fold of the monomer was consistent with an independently built AlphaFold model and with a model based on the honeybee RDL structure (Supplementary Table S1 and Figures S6 and S7; see Section 4 for the limitations of this comparison).

### 3.2. Apo-State Simulations Reveal Largely Conserved Global Dynamics with Localized Differences Between WT and Mutant Receptors

**Figure 2:**
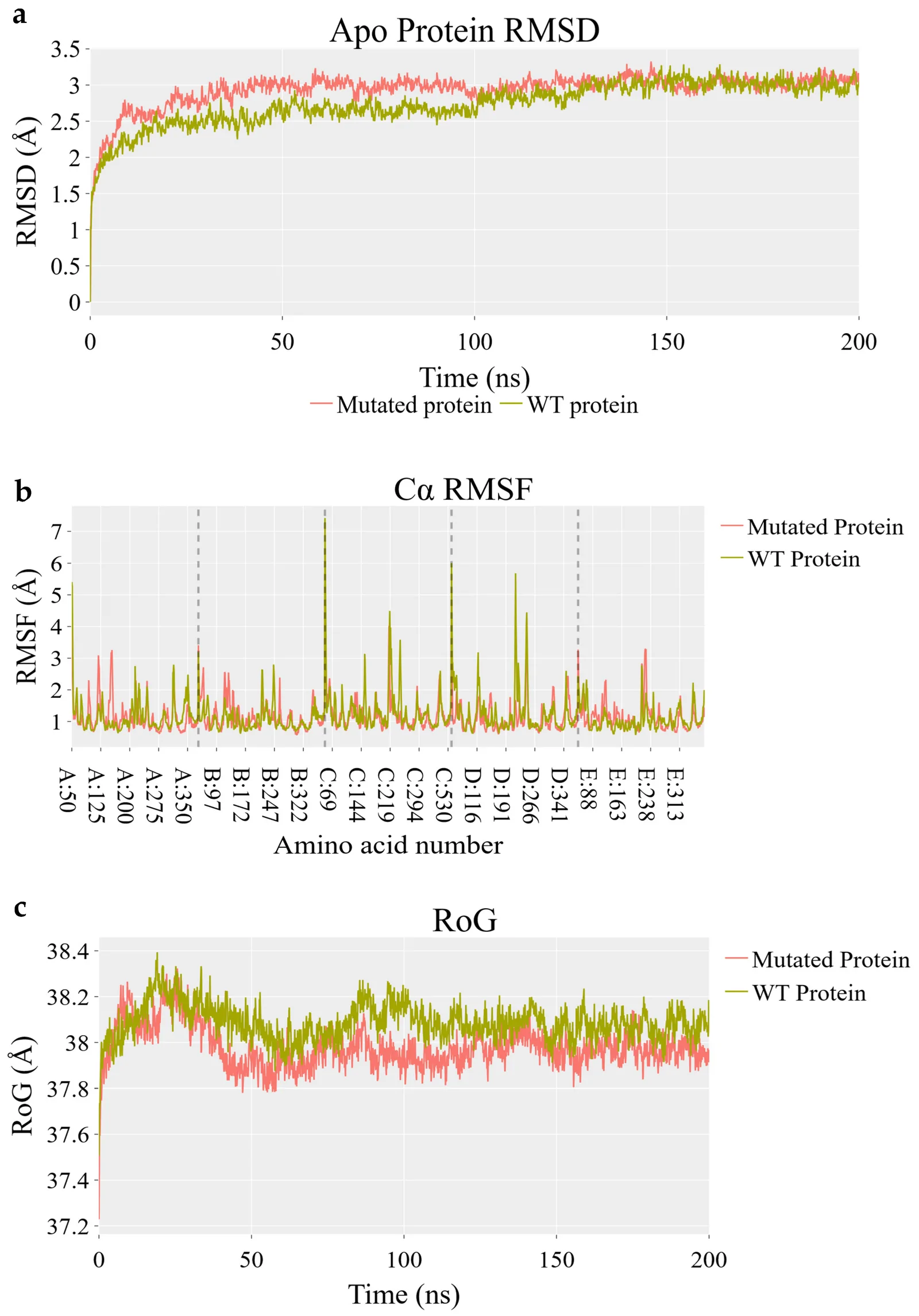

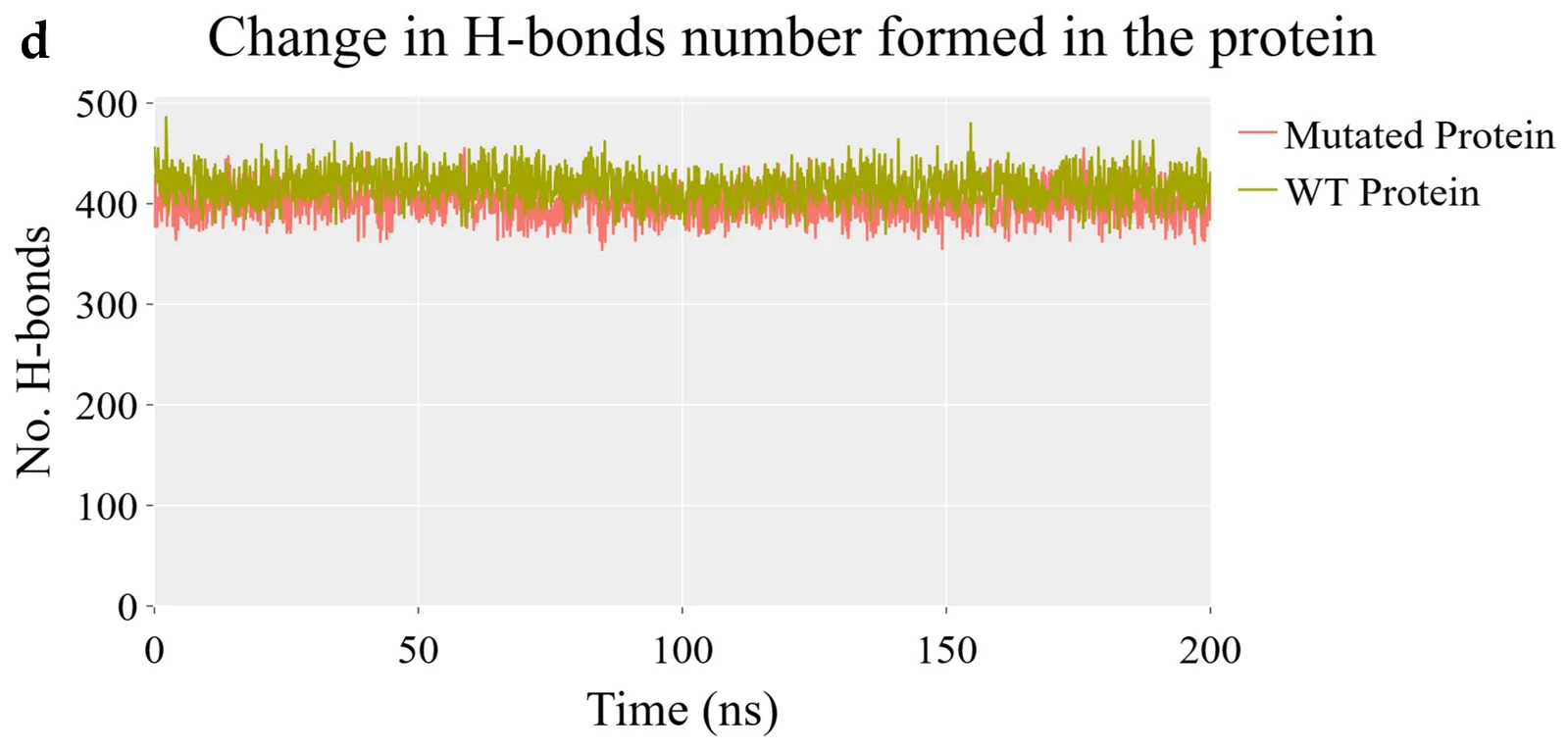
Conformational dynamics of the apo WT and A296S mutant receptors over 200 ns of unrestrained molecular dynamics simulation. Mutant protein, pink/red line; WT protein, olive/green line. (**a**) Backbone root-mean-square deviation (RMSD) as a function of simulation time. (**b**) Per-residue Cα root-mean-square fluctuation (RMSF); dashed vertical lines demarcate chain boundaries (chains A–E), with residue numbers indicated along the *x*-axis. (**c**) Radius of gyration (RoG) as a function of simulation time, reflecting global structural compactness. (**d**) Total number of intramolecular hydrogen bonds within the protein as a function of simulation time. Each apo system was simulated once.

To evaluate whether the A296S substitution alters global receptor stability or dynamics in the unliganded state, 200 ns production simulations were performed for both the WT and mutant apo systems. Each apo system was simulated once. Backbone RMSD indicated that both systems reached comparable structural equilibrium, though with distinct trajectories (Figure 2a). The mutant receptor equilibrated after approximately 50 ns and stabilized at an average RMSD of approximately 3 Å. The WT receptor displayed a biphasic profile, with an average RMSD of 2.6 Å between 50 and 100 ns that increased marginally to approximately 3 Å over the final 100 ns, ultimately converging with the mutant trajectory.

Per-residue flexibility (RMSF) was highly similar overall between the two systems, with deviations confined to specific, chain-dependent regions (Figure 2b). In chain A, the mutant exhibited increased flexibility (>2 Å relative to WT) within residues 118–123 and 150–155. In chain B, the mutant showed elevated fluctuations within residues 117–120 and 128–130, whereas the WT receptor instead displayed local flexibility peaks within residues 214–217 and 244–246. In chain C, elevated flexibility was restricted to the WT receptor, within residues 152–155 and 243–246. In chain D, the WT receptor consistently showed greater local mobility than the mutant within residues 118–122, 214–219, and 223–226. These findings indicate that the A296S substitution does not produce a uniform, system-wide change in flexibility but instead redistributes local dynamics in a chain-specific manner.

Global compactness, assessed via radius of gyration, was nearly identical between the two systems, with mean values of 38.1 Å (WT) and 38.0 Å (mutant) (Figure 2c). A modest reduction in the average number of intramolecular hydrogen bonds was observed in the mutant receptor (400) relative to WT (420) (Figure 2d), suggesting a small, localized effect of the substitution on internal packing rather than a global destabilization of the fold.

Conformational clustering of the apo trajectories using TTClust yielded six representative clusters for the mutant receptor and five for the WT receptor, each used to generate representative structures for subsequent docking.

### 3.3. Docking Analysis Identifies Ligand-Specific Differences in Predicted Binding Affinity and Interaction Architecture

Molecular docking of fipronil and fluralaner against the representative WT and mutant receptor conformations revealed a consistent affinity hierarchy in which fluralaner outperformed fipronil across all sampled receptor states. For the mutant receptor (Table 1), fluralaner top poses ranged from −10.4 to −9.737 kcal/mol, while fipronil top poses ranged from −8.402 to −7.498 kcal/mol. A parallel trend was observed for the WT receptor (Table 2), with fluralaner poses ranging from −9.986 to −8.843 kcal/mol and fipronil poses ranging from −7.201 to −6.937 kcal/mol. The Vina scores were obtained for rigid receptor conformations and were not used to rank the genotypes, because for fluralaner the ordering of WT and A296S after molecular dynamics (Section 3.4 and Section 3.5) differs from that of the docking scores.

**Figure 3:**
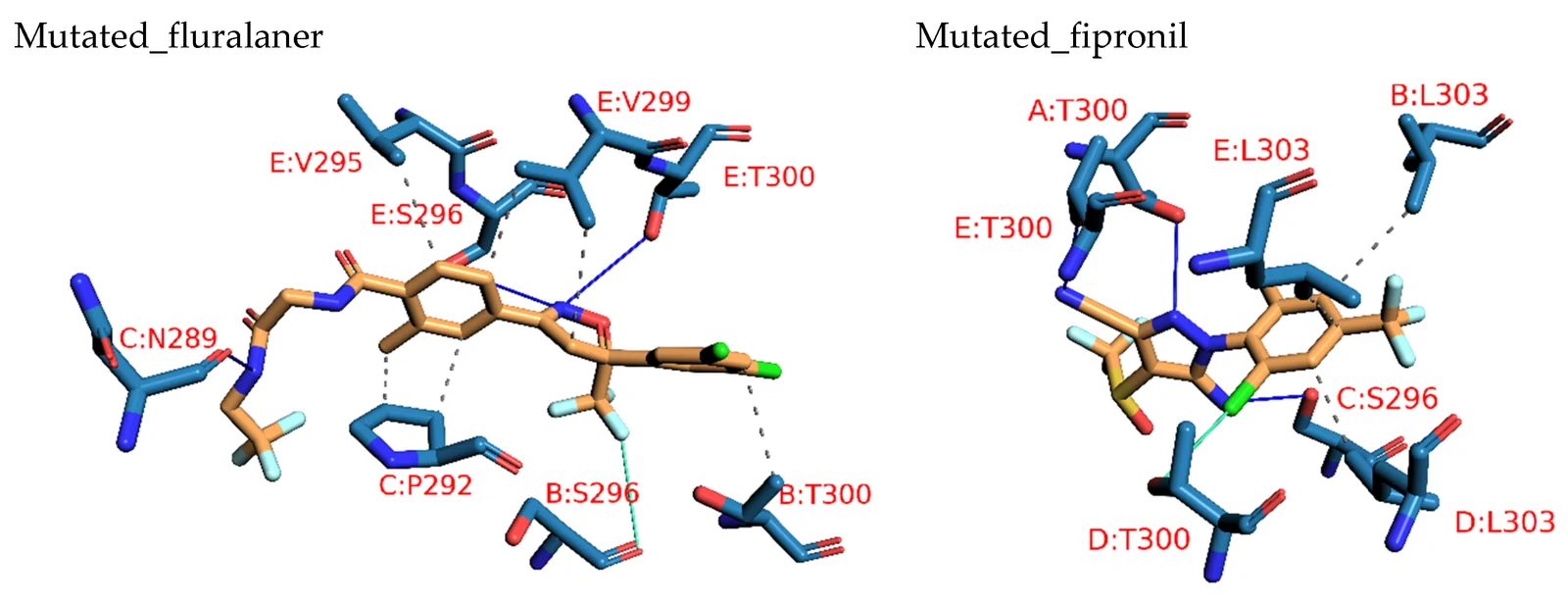
Predicted intermolecular interactions between the A296S mutant receptor and fluralaner (**left**) or fipronil (**right**), based on the highest-affinity docking pose. Interactions are mapped using the Protein–Ligand Interaction Profiler (PLIP): grey dashed lines, hydrophobic contacts; blue solid lines, hydrogen bonds; cyan solid lines, halogen bonds. Ligands are shown as orange sticks and interacting residues as blue sticks, labeled by chain identity and residue name. Other colors in the molecular representation denote the corresponding atom types (e.g., red, oxygen; cyan, carbon; green, halogen atoms).

Interaction profiling of the highest-affinity poses revealed distinct binding architectures for each ligand-receptor combination. In the mutant-fluralaner complex (Figure 3, left), the ligand engaged in an extensive network comprising hydrophobic contacts with B:Thr300, C:Pro292 (two contacts), E:Val295, and E:Val299 (two contacts); hydrogen bonds with C:Asn289, E:Ser296, and E:Thr300; and a halogen bond with B:Ser296. In the mutant-fipronil complex (Figure 3, right), hydrophobic contacts were formed primarily with Leu303 across chains B, D, and E, supplemented by hydrogen bonds with A:Thr300, C:Ser296, and E:Thr300, and a single halogen bond with D:Thr300, indicating a comparatively sparser interaction network than that observed for fluralaner.

**Figure 4:**
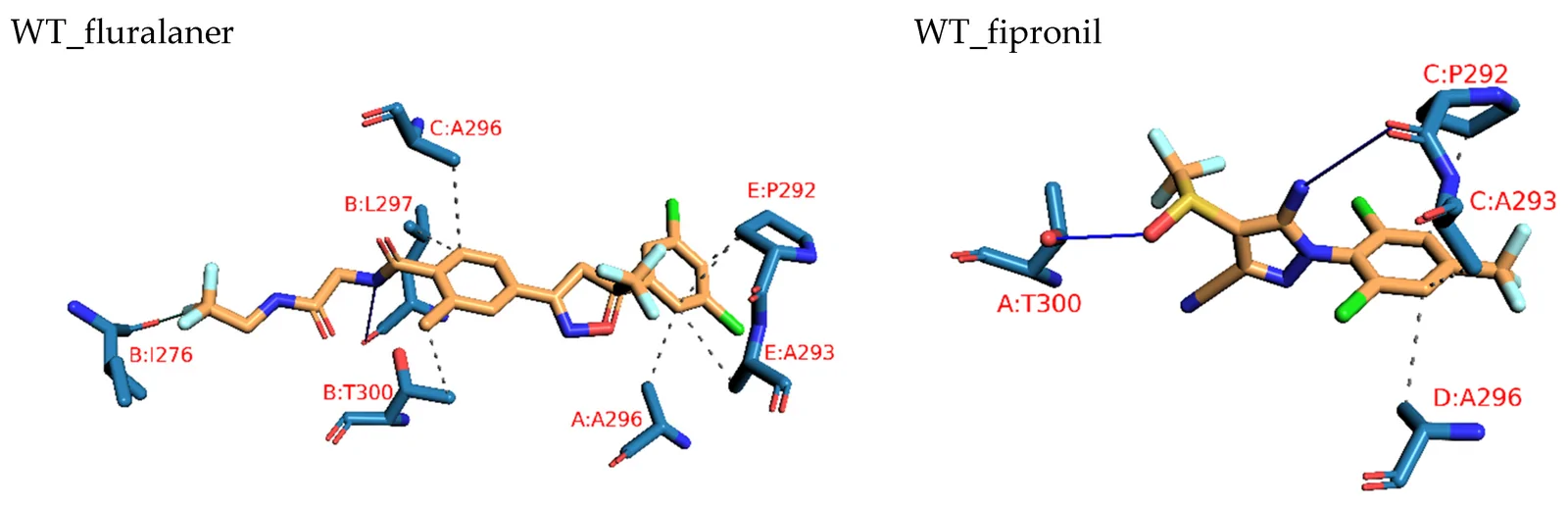
Predicted intermolecular interactions between the wild-type receptor and fluralaner (**left**) or fipronil (**right**), based on the highest-affinity docking pose. Interaction types and rendering conventions are as described for Figure 3.

For the WT receptor, interaction profiling using the Protein–Ligand Interaction Profiler (PLIP) revealed that the WT-fluralaner complex (Figure 4, left) was stabilized by hydrophobic contacts with A:Ala296, B:Leu297, B:Thr300, C:Ala296, E:Pro292 (two contacts), and E:Ala293, together with a hydrogen bond to B:Leu297 and a halogen bond directed toward B:Ile276. The WT-fipronil complex (Figure 4, right) exhibited a more limited interaction profile, comprising hydrophobic contacts with C:Pro292, C:Ala293, and D:Ala296, and hydrogen bonds with A:Thr300 and C:Pro292. Notably, direct engagement of residue 296 by fipronil was observed only in the mutant complex (via C:Ser296), whereas in the WT complex this position contributed solely through Ala296 hydrophobic contact, suggesting that the serine substitution introduces a new polar contact point in the docked fipronil pose; Ser296 also contacts fluralaner in the docked mutant complex (Figure 3). The highest-affinity pose from each combination was carried forward into 200 ns MD simulations to assess binding stability under physiological conditions.

### 3.4. Molecular Dynamics and MM/PBSA Analyses of the A296S Mutant Complexes

#### 3.4.1. Dynamic Behavior

**Figure 5:**
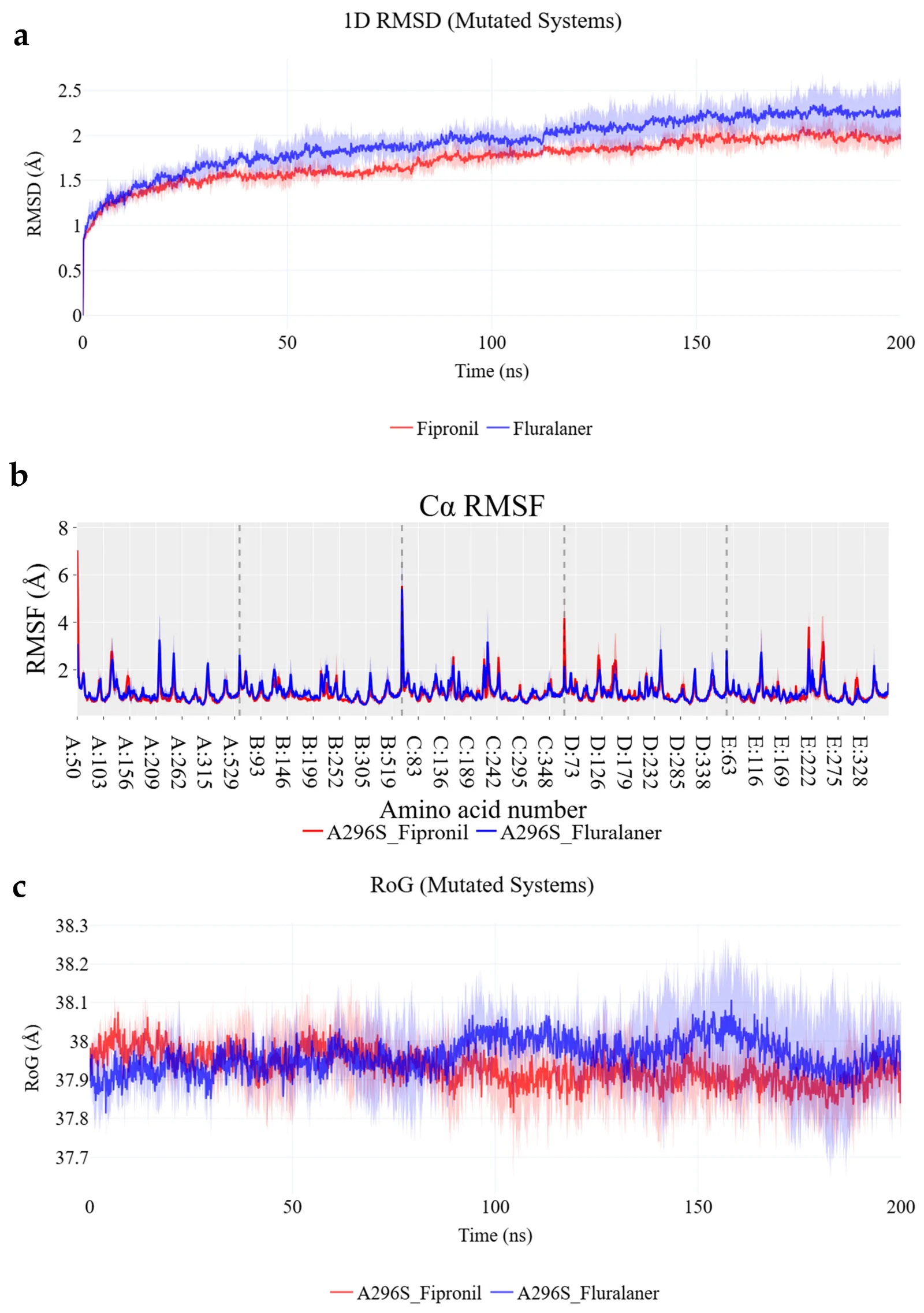

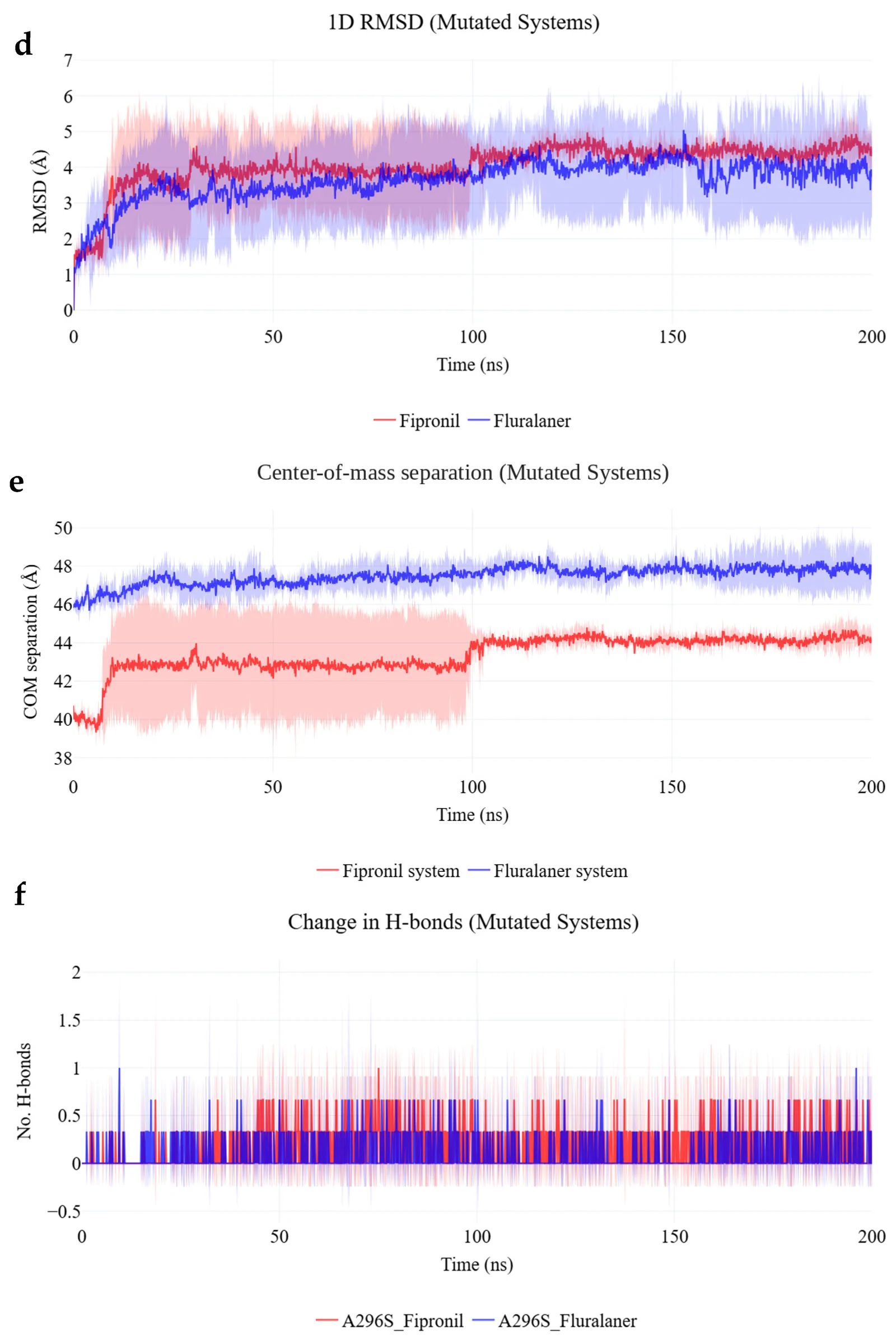

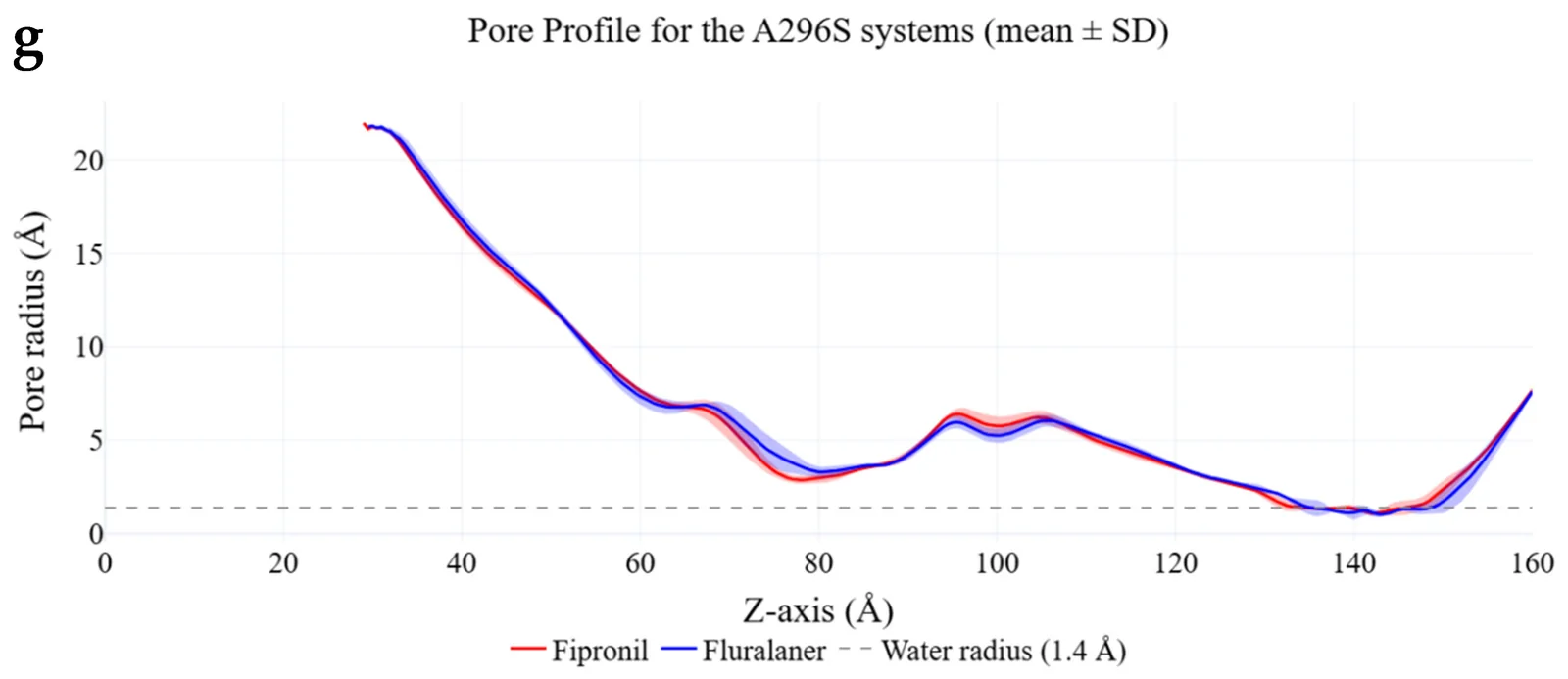
Molecular dynamics simulations of the A296S mutant receptor in complex with fipronil or fluralaner. Mutant protein–fipronil complex, red line; mutant protein–fluralaner complex, blue line. (**a**) Protein backbone RMSD over the 200 ns production trajectory. (**b**) Cα RMSF across the protein sequence. (**c**) Radius of gyration of the protein–ligand complex. (**d**) Ligand RMSD, reflecting conformational stability of the bound ligand within the binding pocket. (**e**) Center-of-mass distance between the ligand and the receptor. (**f**) Number of hydrogen bonds formed between the ligand and the receptor as a function of simulation time. Panels (**a**,**c**–**g**) show the mean of three independent replicas, with shaded bands indicating the standard deviation. (**g**) The profile of pore radius of the A296S mutant receptor in complex with fipronil or fluralaner along the *z*-axis. The mean value over three replicates is shown with standard deviation as shades (mean ± s.d).

Both mutant-ligand complexes displayed comparable receptor backbone dynamics (Figure 5a; mean of three replicas), with RMSD rising gradually to approximately 1.6–1.8 Å by 50 ns and to approximately 2.0–2.3 Å by the end of the simulation. This was corroborated by similarly low RMSF profiles for both complexes, generally below 2 Å (Figure 5b). Radius of gyration values remained closely matched for the first 150 ns before diverging modestly, with the fipronil complex decreasing slightly and the fluralaner complex increasing slightly over the final 50 ns (Figure 5c). Ligand RMSD values were broadly comparable between the two compounds over the final 150 ns (approximately 3.5–4.5 Å, with wide replica-to-replica variability; Figure 5d), indicating that both ligands remained in the binding region, although fluctuations of this size do not demonstrate a single well-defined pose. The ligand-receptor center-of-mass separation of fluralaner remained at approximately 46–48 Å throughout the simulation, whereas that of fipronil increased from approximately 40 Å to approximately 43–44 Å within the first 10 ns and then remained approximately constant (Figure 5e). Hydrogen bonding was more frequent between fipronil and the mutant receptor than between fluralaner and the mutant receptor (Figure 5f), consistent with the polar contacts identified during docking. The pore radius profiles of the A296S complexes (Figure 5g) were lower than those of the corresponding WT complexes (Figure 7g) over part of the *z*-axis. At z = 78 Å, the mean pore radius was 2.877 Å for the fipronil complex and 3.593 Å for the fluralaner complex in the A296S receptor, compared with 5.793 Å and 5.457 Å, respectively, in the WT receptor, whereas the narrowest region (near z = 140 Å) was similar in both genotypes. This genotype difference is compatible with, but does not by itself demonstrate, inward orientation of the Ser296 hydroxyl groups, and the z-position of residue 296 is not annotated in the profiles. The profiles describe the geometry of the modeled ligand-bound complexes only and do not show ion permeation, conductance, or functional pore occlusion.

#### 3.4.2. Binding Energetics

**Figure 6:**
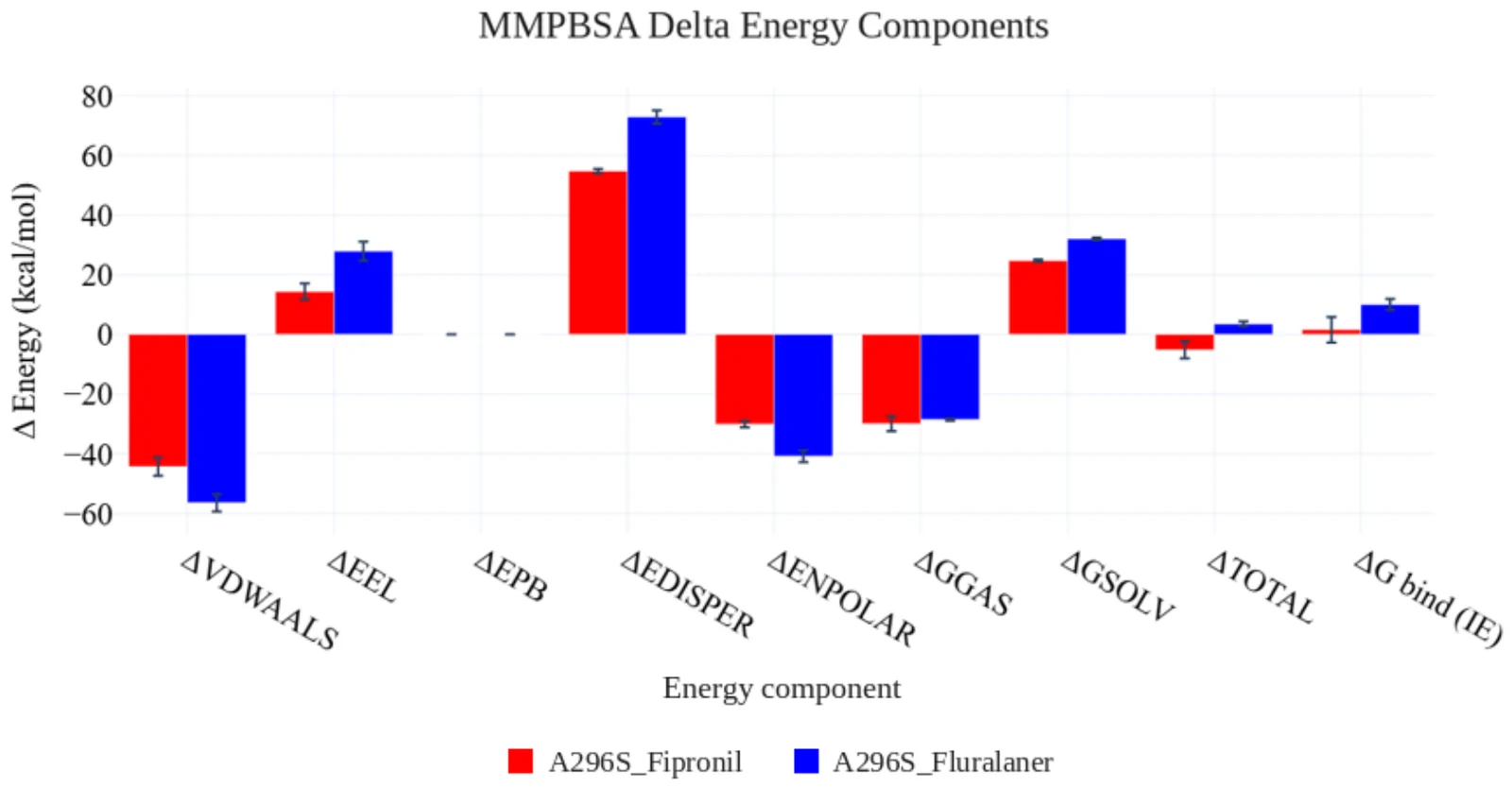
MM/PBSA binding free-energy components for the A296S mutant receptor in complex with fipronil (red bars) or fluralaner (blue bars). Energy terms are decomposed into van der Waals (Δvdwaals), electrostatic (Δeel), polar solvation (Δepb), Dispersion (Δedisper), non-polar solvation (Δenpolar), total gas-phase (Δggas), total solvation (Δgsolv), total enthalpy before the interaction-entropy term (Δtotal), and total free energy with entropy (ΔG bind(IE)) contributions. Values represent means across three replicas; error bars denote standard deviation.

For the mutant-fipronil complex, the van der Waals term (ΔevdW, −44.280 ± 3.090 kcal/mol) and the non-polar (cavity) term (Δenpolar, −30.060 ± 1.073 kcal/mol) were favorable, whereas the electrostatic term (Δeel, +14.363 ± 2.686 kcal/mol) and the dispersion term (Δedisper, +54.780 ± 0.727 kcal/mol) were unfavorable; the polar solvation term (Δepb) was approximately zero with the heterogeneous-dielectric implicit-membrane protocol used here (Figure 6). The resulting ΔG including interaction entropy was +1.590 ± 4.316 kcal/mol (mean ± SD across three replicas), which is not favorable within the uncertainty. For the mutant-fluralaner complex, ΔevdW (−56.440 ± 2.882 kcal/mol) and Δenpolar (−40.827 ± 1.946 kcal/mol) were favorable, whereas Δeel (+27.887 ± 3.161 kcal/mol) and Δedisper (+72.883 ± 2.180 kcal/mol) were unfavorable, giving a ΔG including interaction entropy of +10.023 ± 1.848 kcal/mol, which is more unfavorable than that of fipronil in the mutant receptor.

Per-residue decomposition of the mutant-fipronil complex revealed no residues contributing more than −1 kcal/mol to ligand binding (Figure S1). For the mutant-fluralaner complex, two residues exceeded this threshold slightly: Val295 (−1.076 ± 0.514), and Val299 (−1.218 ± 0.254) (Figure S1), indicating a conserved high-contribution residue Val299 in both WT and A296S mutant. Ser296 itself made a small positive (unfavorable) per-residue contribution to both ligands in the mutant (Figure S1), so the substituted residue did not act as a favorable energetic anchor for fipronil in these simulations. The enthalpic Δtotal over the last 100 ns (Figure S2) fluctuated around approximately −5 kcal/mol for fipronil and approximately +4 kcal/mol for fluralaner without a pronounced trend.

### 3.5. Molecular Dynamics and MM/PBSA Analyses Reveal Divergent Fipronil and Fluralaner Stability in the Wild-Type Receptor

#### 3.5.1. Dynamic Behavior

**Figure 7:**
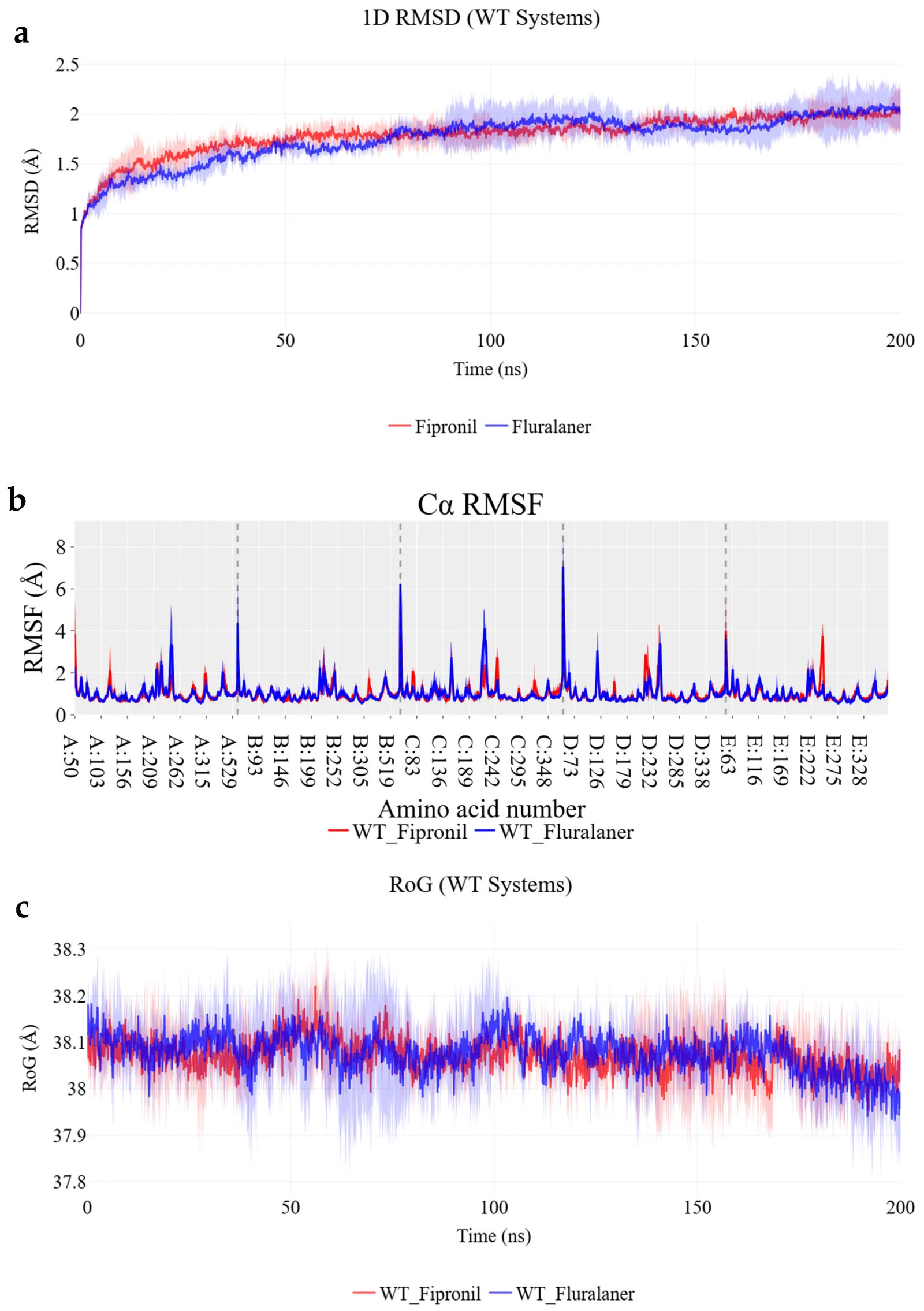

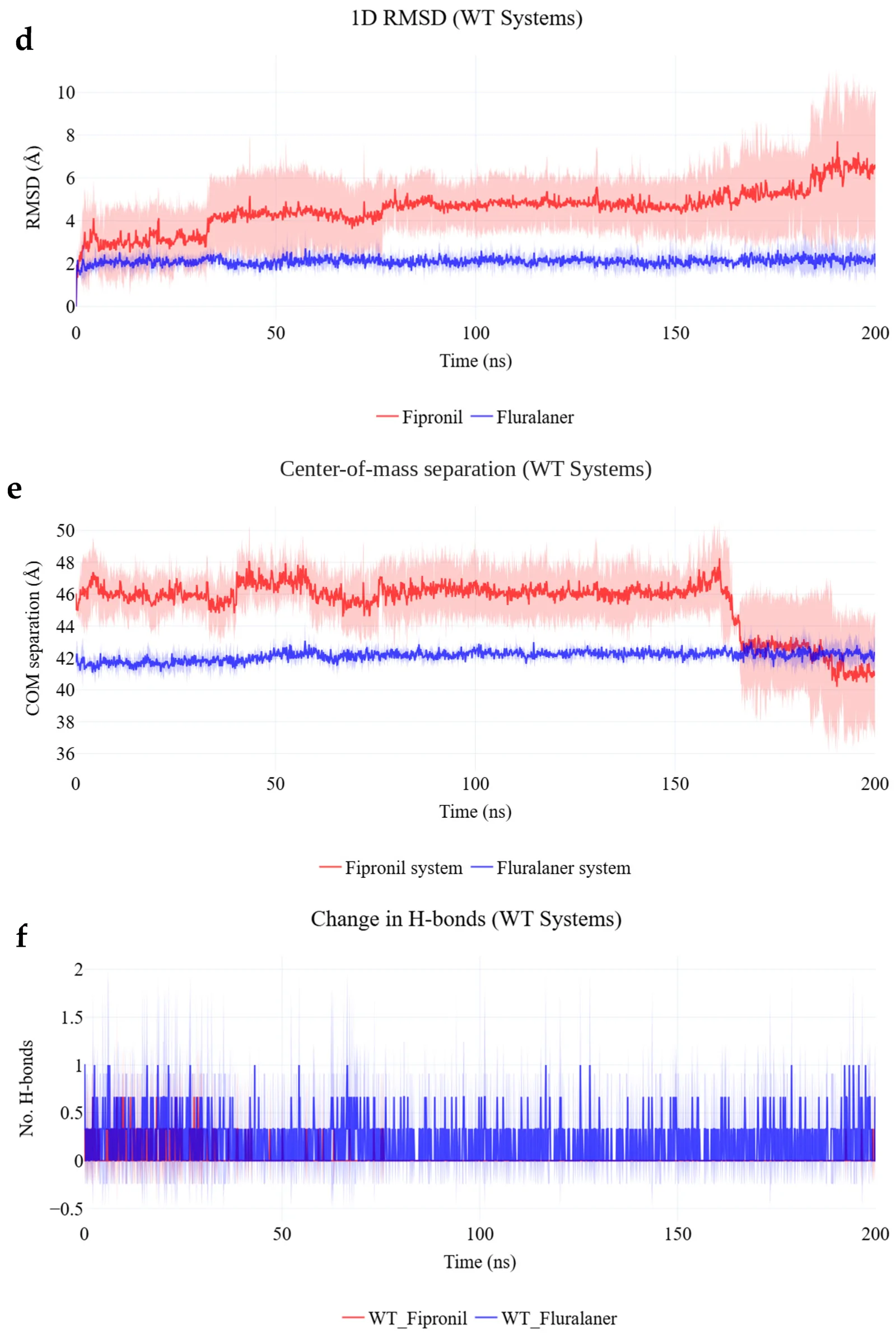

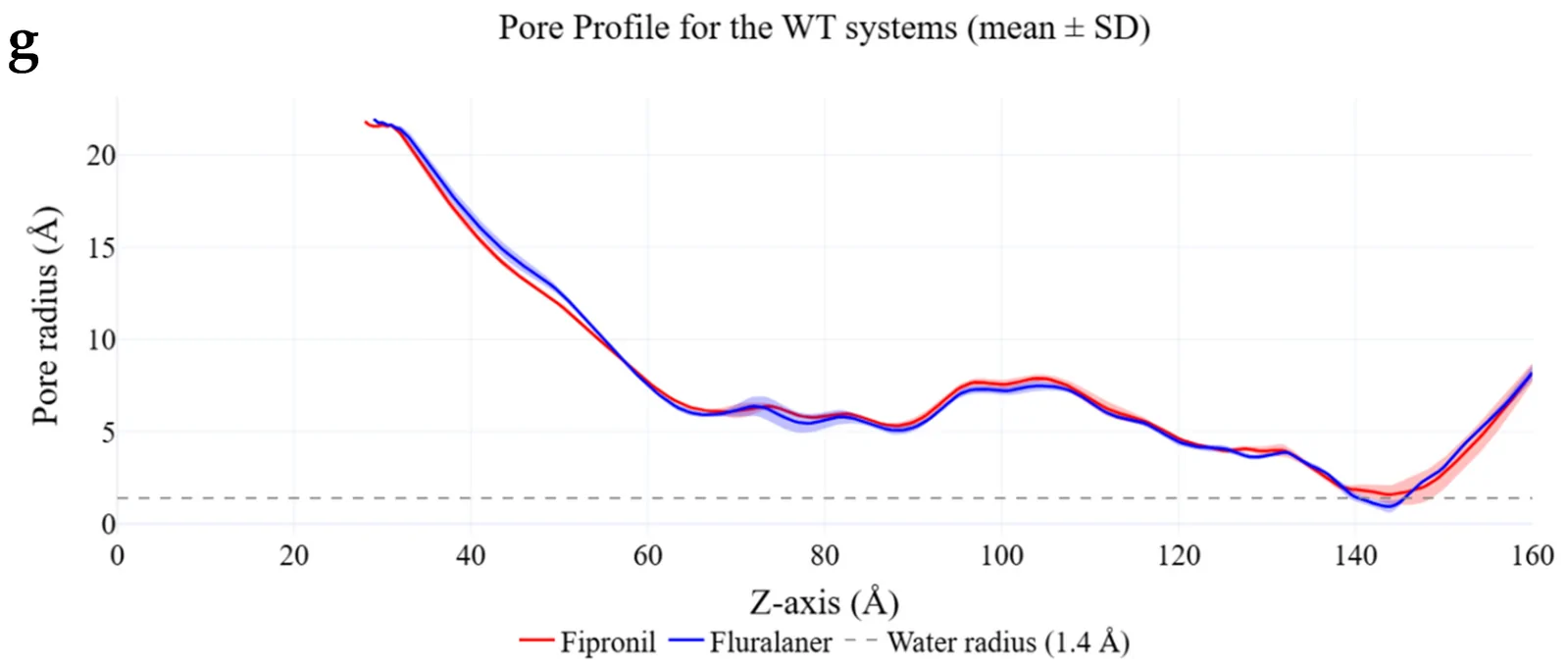
Molecular dynamics simulations of the wild-type receptor in complex with fipronil or fluralaner. WT protein–fipronil complex, red line; WT protein–fluralaner complex, blue line. (**a**) Protein backbone RMSD. (**b**) Cα RMSF across the protein sequence. Vertical dashed lines indicate the boundaries between adjacent receptor subunits (chains A–E). (**c**) Radius of gyration of the protein–ligand complex. (**d**) Ligand RMSD over the simulation trajectory. (**e**) Center-of-mass distance between the ligand and the receptor. (**f**) Number of hydrogen bonds formed between the ligand and the receptor. Panels (**a**,**c**–**g**) show the mean of three independent replicas, with shaded bands indicating the standard deviation. (**g**) The profile of pore radius of the wild-type receptor in complex with fipronil or fluralaner along the *z*-axis. The mean value over three replicates is shown with standard deviation as shades (mean ± s.d).

Backbone RMSD trends were similar between the two WT complexes, with the fluralaner complex maintaining marginally lower values, and both systems reaching a stable average during the final 50 ns (Figure 7a). RMSF and radius of gyration profiles were likewise comparable between complexes, indicating preserved global receptor compactness (Figure 7b,c). In marked contrast, ligand RMSD diverged substantially between the two compounds (Figure 7d). Fluralaner remained stable throughout the simulation, with an average RMSD of approximately 2 Å. The mean ligand RMSD of fipronil increased from approximately 3 Å to approximately 5 Å during the first 100 ns and to approximately 6–7 Å over the final 40 ns, with a wide replica-to-replica spread (the shaded band reaches approximately 10 Å at the end). Over the final 40 ns, the fipronil-receptor center-of-mass separation decreased from approximately 46 Å to approximately 42 Å, with a wide shaded band (Figure 7e), consistent with a shift in the ligand relative to its initial position. Fluralaner maintained a stable separation of approximately 42 Å throughout and formed hydrogen bonds with the receptor more frequently than fipronil (Figure 7f). The pore radius profiles of the two WT complexes were closely similar (Figure 7g), with the largest difference near z = 140 Å, where the minimum radius was slightly smaller in the fluralaner complex; these profiles describe the modeled geometry only. Together, these observations suggest that within the WT receptor, fluralaner occupies a comparatively more stable binding mode than fipronil over the simulated timescale. In the A296S receptor, the mean fipronil ligand RMSD at the end of the simulations (approximately 4.5 Å) was lower than in the WT receptor (approximately 6–7 Å), but the replica bands overlap, and this was not accompanied by a more favorable free-energy estimate (Section 3.4.2).

#### 3.5.2. Binding Energetics

**Figure 8:**
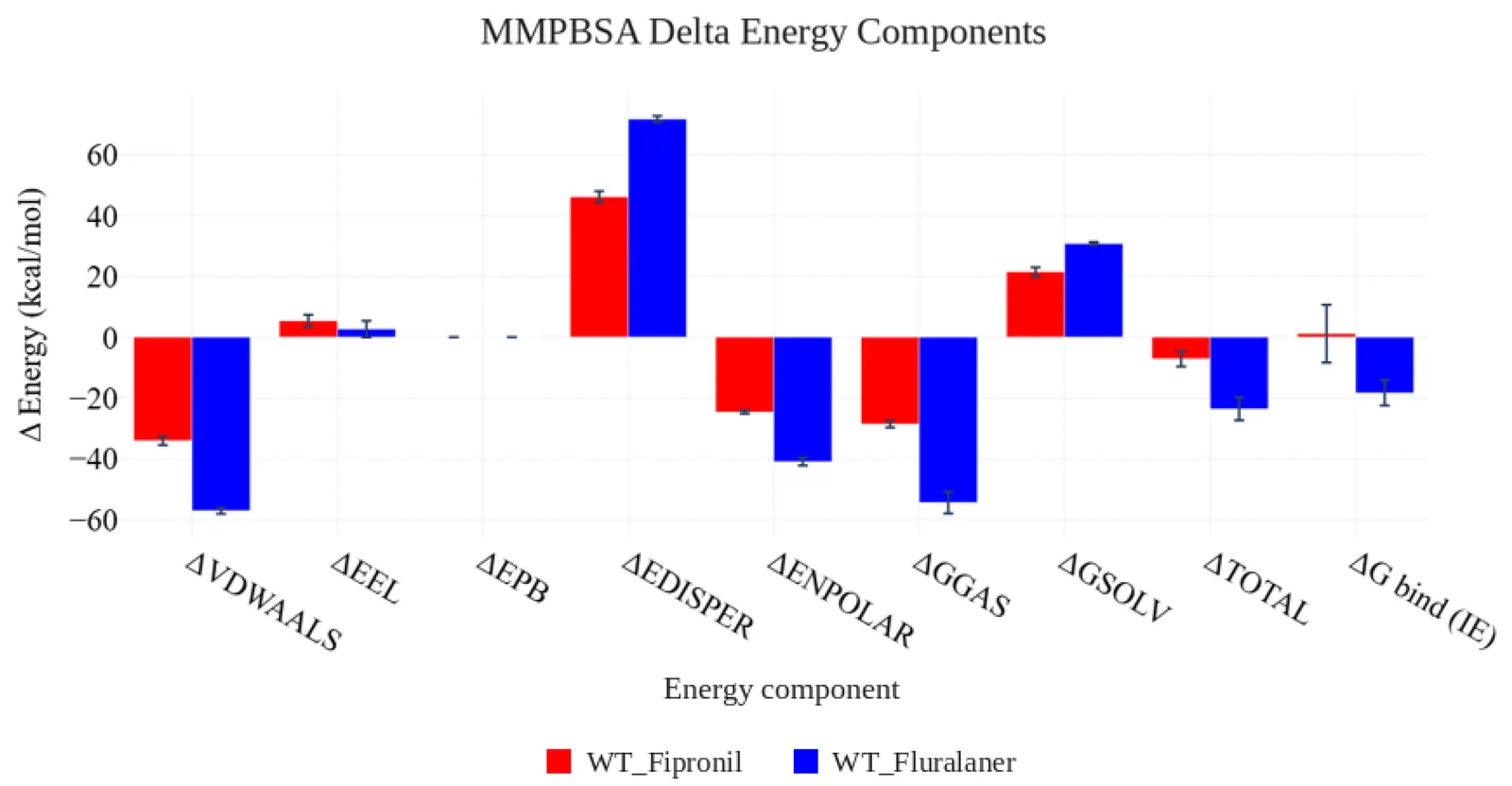
MM/PBSA binding free-energy components for the wild-type receptor in complex with fipronil (red bars) or fluralaner (blue bars). Energy terms and error bars are as described for Figure 6.

MM/PBSA analysis of the WT complexes confirmed a substantially more favorable binding energy for fluralaner relative to fipronil (Figure 8). The WT-fluralaner complex had favorable van der Waals (ΔEvdW, −57.030 ± 1.005 kcal/mol) and non-polar (ΔEnpolar, −40.907 ± 1.276 kcal/mol) terms, which together outweighed the electrostatic (ΔEel, +2.633 ± 2.767 kcal/mol) and dispersion (ΔEdisper, +71.690 ± 1.025 kcal/mol) terms. The ΔG including interaction entropy was −18.317 ± 4.208 kcal/mol. The WT-fipronil complex showed weaker van der Waals (−34.000 ± 1.433 kcal/mol) and non-polar (−24.647 ± 0.480 kcal/mol) terms and less favorable electrostatic (+5.410 ± 1.947 kcal/mol) and dispersion (+46.120 ± 1.880 kcal/mol) terms; its ΔG including interaction entropy was +1.177 ± 9.480 kcal/mol, which is not distinguishable from zero given the replica spread.

Per-residue decomposition of the WT-fipronil complex showed no residue contribution exceeding −1 kcal/mol (Figure S3). By contrast, the WT-fluralaner complex showed only two residues surpassing this threshold, including Leu297 (−2.663 ± 0.208), and Val299 (−1.203 ± 0.093) (Figure S3), reflecting a slightly broader and energetically deeper interaction network relative to fipronil. Time-resolved binding free-energy analysis (Figure S4) confirmed that both compounds maintained stable interactions with the WT receptor across the last 100 ns of simulation, with fipronil consistently exhibiting a less favorable binding profile than fluralaner throughout the trajectory.

#### 3.5.3. Comparison Across Genotypes

Considered together, the replicated energetic analyses do not support Ser296 acting as a favorable energetic anchor for fipronil in the mutant receptor. The free-energy estimate for fipronil was similar in the WT (+1.177 ± 9.480 kcal/mol) and A296S (+1.590 ± 4.316 kcal/mol) receptors, and Ser296 made a small unfavorable per-residue contribution in both mutant complexes (Figure S1). The mean fipronil ligand RMSD at the end of the A296S simulations was nevertheless lower than in the WT simulations, although the replica bands overlap. For fluralaner, the free-energy estimate changed from −18.317 ± 4.208 kcal/mol in the WT receptor to +10.023 ± 1.848 kcal/mol in the A296S receptor, and the per-residue profile changed: Leu297 (−2.663 ± 0.208 kcal/mol in the WT complex) was not a major contributor in the mutant, whereas Val295 and Val299 contributed modestly in the mutant (Figures S1 and S3). Part of this difference may reflect different starting positions of the docked fluralaner poses (selected by docking score from different receptor conformations), as suggested by the different center-of-mass separations (approximately 42 Å in the WT complex and 46–48 Å in the mutant complex; Figure 5e and Figure 7e), rather than a direct effect of residue 296 alone. In this model, A296S is therefore associated with a larger change in the predicted energetics of fluralaner than of fipronil. These findings concern modeled equilibrium binding only.

## 4. Discussion

The present study addresses how a single, conservative substitution at the 2′ position of the M2 helix, A296S, may differentially affect the modeled recognition of two structurally distinct insecticides, fipronil and fluralaner, that act at overlapping transmembrane sites of the *An*. *gambiae* RDL receptor. Through an integrated pipeline of homology modeling, docking, molecular dynamics (MD; three replicas for each ligand-bound system), and MM/PBSA analysis with an interaction-entropy term, we find that A296S does not function as a uniform steric or electrostatic barrier. Instead, it locally reconfigures the physicochemical environment of the antagonist-binding cavity in a manner that is asymmetrically exploited by the two ligands. These results should be read as evidence of a ligand-dependent energetic landscape, not as a demonstration that A296S confers or removes functional insecticide resistance, a distinction that is central to the interpretation developed below.

Apo-protein simulations indicate that A296S does not compromise the global architecture of the pentameric channel. In single 200 ns apo trajectories per genotype, wild-type (WT) and mutant receptors embedded in a POPC bilayer reached comparable backbone RMSD plateaus, closely matched radii of gyration, and largely overlapping RMSF profiles, with fluctuation differences confined to discrete, non-contiguous loop segments rather than to the transmembrane core. This pattern is consistent with residue 296 governing local channel-lining chemistry within M2 rather than playing a structural role in tertiary packing of the extracellular or intracellular domains [8,58]. The HOLE profiles of the ligand-bound complexes show a smaller pore radius in the A296S complexes over part of the *z*-axis (Figure 5g and Figure 7g), compatible with an inward orientation of the serine hydroxyl group, an arrangement previously modeled to alter steric and polar properties at the classical antagonist site in African Anopheles populations [10]. Because these profiles are geometric descriptions of modeled complexes and no ion-permeation, conductance, or gating simulations were performed, they should be regarded as a plausible geometric consequence rather than a demonstrated constriction of the functional conduction pathway. Such localized, non-destabilizing remodeling is consistent with the persistence of the A296S allele in field populations despite documented fitness costs associated with Rdl-based resistance in *An. gambiae* [59].

Docking and MM/PBSA analyses reveal an energetic asymmetry between the two ligands. In the WT receptor, fluralaner had the more favorable free-energy estimate (−18.3 ± 4.2 kcal/mol), dominated by van der Waals and non-polar terms with a comparatively small electrostatic term, consistent with the characterization of isoxazolines as antagonists whose potency depends on hydrophobic complementarity within a broader transmembrane cavity spanning M1 to M3 [60,61]. Fipronil showed weaker van der Waals and non-polar terms and an estimate near zero (+1.2 ± 9.5 kcal/mol). In the A296S receptor, the fipronil estimate was essentially unchanged (+1.6 ± 4.3 kcal/mol), whereas the fluralaner estimate became unfavorable (+10.0 ± 1.8 kcal/mol). The polar solvation term plotted in Figure 6 and Figure 8 was approximately zero for all four complexes with the implicit-membrane protocol used, so the differences arise from the van der Waals, electrostatic, dispersion, and non-polar terms. Per-residue decomposition did not identify Ser296 as a favorable contributor for either ligand; its contribution was small and positive in both mutant complexes. Stabilization of fluralaner was spread over many residues in both genotypes, with individual contributions mostly between −0.5 and −1.3 kcal/mol, the exception being Leu297 (−2.7 kcal/mol) in the WT complex (Figures S1 and S3).

Complementary MD trajectories of the ligand-bound complexes show a further difference. In the replicated WT simulations, the fluralaner ligand RMSD remained near 2 Å, whereas the mean fipronil ligand RMSD increased progressively to approximately 6–7 Å with large replica-to-replica variability. In the A296S receptor, both ligands sampled broadly similar ligand RMSD values (approximately 3.5–4.5 Å) over the final 150 ns, with more frequent hydrogen bonding recorded for fipronil. The lower fipronil ligand RMSD in the mutant therefore coincided with an unchanged, not a more favorable, free-energy estimate, and ligand RMSD alone should not be read as evidence of tighter binding.

The absence of a detectable difference in the fipronil free-energy estimate between the WT and A296S models is compatible with the limited and variable cross-resistance to fipronil reported for A296S/A302S, although the large uncertainty of the fipronil estimates (SD up to 9.5 kcal/mol) limits what can be concluded. Equilibrium binding estimates are not predictions of functional sensitivity or resistance. Resistance at RDL is a pharmacological property that depends on state-dependent channel block, gating kinetics, and a ligand’s ability to stabilize or be excluded from specific conformational states (closed, open, desensitized) across repeated activation [2], none of which were modeled here; all simulations started from a single receptor conformational state inherited from the template. Interpreted cautiously, the data are not inconsistent with the established role of A296S in cyclodiene resistance and its limited effect on fipronil [8,13].

These findings should be set against the pharmacological distinction between fiproles and isoxazolines at RDL. Structural and functional work indicates that isoxazolines occupy a transmembrane cavity that partially overlaps with, but is not identical to, the canonical noncompetitive-antagonist pocket historically defined by cyclodiene-resistance mutations, contributing to the generally limited cross-resistance observed between older and newer chemistries [7,60]. The small per-residue contribution of Ser296 itself to fluralaner binding is compatible with that separation, but the less favorable fluralaner free-energy estimate in the A296S model is not obviously so. It may reflect indirect effects of the substitution on local pocket geometry, non-equivalent docked starting poses, or limitations of the method. Phenotypic evidence for A296S and isoxazolines is limited, and a recent study suggests that A296S can influence fluralaner sensitivity [7], so the present result is inconclusive with respect to fluralaner resistance. Published work is consistent with external transmembrane residues, rather than the classical 2′ pore-lining position, dictating much of fluralaner’s mode of action [61], and with evidence that a distinct, second-site RDL mutation outside the 2′ position governs isoxazoline and meta-diamide resistance independently of A296S [7,62]. Recent cryo-EM structures of insect RDL receptors have begun to resolve how the M1 to M3 transmembrane bundle accommodates multiple, only partially overlapping allosteric sites [3], providing a structural framework within which the present homology-based predictions can be interpreted, while underscoring that direct structural validation for the *An. gambiae* receptor itself remains unavailable.

More broadly, the limited and variable cross-resistance reported between A296S/A302S and fipronil across insect species and genetic backgrounds [6,12,13,63] is compatible with a model in which equilibrium binding interactions are insufficient on their own to predict functional resistance phenotypes.

From an applied standpoint, the present results do not allow a conclusion on whether isoxazolines retain activity against A296S-carrying populations: the modeled fluralaner free energy was less favorable in the A296S receptor, whereas published phenotypic evidence is limited and was not examined here. This question requires experimental validation and could be influenced by metabolic resistance or additional target-site mechanisms. Residues outside the 2′ position can also modify susceptibility [6,7], so other RDL substitutions may interact with A296S and change the predicted interaction landscape. The data argue against treating the 2′ alanine-to-serine substitution as a generic resistance determinant whose consequences can be inferred from a single chemical class. Fipronil and fluralaner are case-study compounds, and the results should not be generalized to all phenylpyrazoles or isoxazolines. Candidate compounds could be evaluated for their dependence on residue 296; future candidates should be explicitly evaluated for their dependence on residue 296 and on the balance of electrostatic versus hydrophobic contacts, since these appear to be the molecular features most sensitive to this resistance allele.

This study has the usual boundaries of a computational investigation. The receptor is a homology model, and a supplementary comparison with AlphaFold and honeybee-based models agrees on overall fold, although pocket-level agreement was not assessed. Ligand-bound systems were run in three replicas, and MM/PBSA with interaction entropy is best read as a relative, qualitative guide [64]. Gating and ion permeation were not simulated, so pore profiles describe geometry only. Only A296S was examined, with fipronil and fluralaner as case studies. Electrophysiology or binding assays would test these predictions, which we offer as hypotheses for that work, and we welcome such validation.

Taken together, these findings indicate that, in this model, A296S is associated with ligand-specific changes in the interaction landscape of the RDL antagonist pocket, with a larger predicted effect on fluralaner than on fipronil, a distinction addressed further in the Conclusions below.

## 5. Conclusions

In the modeled RDL receptor, the A296S substitution is associated with ligand-specific changes in the antagonist-binding pocket. Rather than acting as a universal determinant, the mutation did not detectably change the weak MM/PBSA free-energy estimate for fipronil, and Ser296 did not act as a favorable energetic anchor, whereas the estimate for fluralaner shifted from favorable in the WT receptor (−18.3 ± 4.2 kcal/mol) to unfavorable in the A296S receptor (+10.0 ± 1.8 kcal/mol). In the WT receptor, fipronil showed greater positional mobility than fluralaner, which remained stable.

These findings suggest that a single conservative pore-lining substitution can produce divergent molecular consequences for chemically distinct insecticides occupying partially overlapping binding environments. This differential remodeling of electrostatic and hydrophobic contacts is compatible with the variable phenotypic effects reported for A296S on fipronil susceptibility; for isoxazolines, phenotypic evidence is limited, and the present fluralaner result is inconclusive with respect to resistance. These computational analyses characterize the equilibrium interaction landscape of a homology model that has not been validated experimentally and should not be read as direct measures of functional resistance, which depends on state-dependent channel block, gating kinetics, and ligand efficacy across multiple conformational states.

By resolving residue-level energetic contributions, this study offers atomistic insight into how A296S differentially shapes recognition of structurally distinct insecticides at the RDL pocket. The findings do not establish whether isoxazolines retain activity against A296S-carrying *An*. *gambiae* populations, which requires electrophysiological or phenotypic testing, but they may help inform structure-guided efforts to evaluate dependence on the classical 2′ pore-lining residue in future RDL-targeting compounds. Fipronil and fluralaner are case-study compounds and not definitive representatives of their classes. More broadly, the results indicate that resistance-associated substitutions must be interpreted within residue-specific ligand interaction networks rather than treated as generic determinants of susceptibility, offering a starting point for understanding cross-resistance between fiprole and isoxazoline chemotypes at this locus.

## Figures and Tables

**Table 1:** Predicted binding affinities of fluralaner and fipronil docked against representative conformations of the A296S mutant receptor. Docking was performed against six representative receptor conformations (C1–C6, derived from TTClust cluster analysis of the 200 ns apo-mutant trajectory) using AutoDock Vina (exhaustiveness = 16). For each complex, binding affinities (kcal/mol) are reported for the nine top-ranked poses (Pose 1–Pose 9), ranked in descending order of predicted affinity.

Complex	Pose 1	Pose 2	Pose 3	Pose 4	Pose 5	Pose 6	Pose 7	Pose 8	Pose 9
Fluralaner_C3	−10.4	−10.35	−10.26	−10.24	−10.22	−10.16	−10.14	−9.978	−9.973
Fluralaner_C1	−10.16	−10.09	−9.985	−9.927	−9.897	−9.783	−9.711	−9.682	−9.639
Fluralaner_C6	−10.07	−10	−9.965	−9.955	−9.891	−9.855	−9.811	−9.713	−9.567
Fluralaner_C2	−9.894	−9.893	−9.563	−9.544	−9.357	−9.326	−9.215	−9.201	−9.139
Fluralaner_C5	−9.894	−9.758	−9.709	−9.401	−9.32	−9.301	−9.25	−9.241	−9.117
Fluralaner_C4	−9.737	−9.715	−9.685	−9.63	−9.576	−9.552	−9.532	−9.509	−9.463
Fipronil_C6	−8.402	−8.401	−7.907	−7.904	−7.834	−7.678	−7.355	−7.352	−7.292
Fipronil_C1	−8.277	−8.275	−8.271	−8.235	−8.213	−8.208	−8.077	−8.023	−8.022
Fipronil_C2	−8.107	−8.103	−8.073	−8.072	−8.053	−8.044	−7.9	−7.847	−7.805
Fipronil_C3	−8.085	−8.078	−7.78	−7.765	−7.7	−7.679	−7.572	−7.514	−7.286
Fipronil_C4	−7.88	−7.877	−7.775	−7.693	−7.689	−7.627	−7.591	−7.527	−7.407
Fipronil_C5	−7.498	−7.489	−7.453	−7.438	−7.365	−7.354	−7.336	−7.286	−7.272

**Table 2:** Predicted binding affinities of fluralaner and fipronil docked against representative conformations of the wild-type receptor. Docking parameters and pose ranking follow those described for Table 1, using representative conformations derived from the 200 ns apo-WT trajectory.

Complex	Pose 1	Pose 2	Pose 3	Pose 4	Pose 5	Pose 6	Pose 7	Pose 8	Pose 9
Fluralaner_C3	−9.986	−9.978	−9.59	−9.531	−9.25	−8.801	−8.783	−8.683	−8.632
Fluralaner_C1	−9.207	−9.163	−8.927	−8.607	−8.565	−8.486	−8.461	−8.423	−8.412
Fluralaner_C5	−9.189	−9.172	−9.073	−9.006	−8.994	−8.973	−8.963	−8.876	−8.866
Fluralaner_C4	−8.962	−8.953	−8.863	−8.777	−8.737	−8.663	−8.573	−8.537	−8.51
Fluralaner_C2	−8.843	−8.803	−8.748	−8.745	−8.654	−8.597	−8.561	−8.548	−8.505
Fipronil_C4	−7.201	−7.184	−7.106	−7.086	−7.026	−7.016	−6.968	−6.966	−6.925
Fipronil_C5	−7.197	−7.185	−7.16	−7.155	−7.075	−7.068	−6.862	−6.823	−6.684
Fipronil_C3	−7.034	−7.032	−7.01	−6.722	−6.696	−6.694	−6.688	−6.653	−6.639
Fipronil_C2	−6.979	−6.96	−6.678	−6.655	−6.652	−6.636	−6.631	−6.614	−6.595
Fipronil_C1	−6.937	−6.925	−6.918	−6.915	−6.913	−6.812	−6.792	−6.691	−6.679

## Data Availability

Data supporting this study are included within the article and/or its Supplementary Materials. Representative structures of the WT and A296S receptor models with the top-ranked docked ligand poses can be provided by the corresponding author on request.

## Supplementary Materials

The following supporting information can be downloaded at https://www.mdpi.com/article/10.3390/biology15212011/s1. Figure S1: Per-residue MM/PBSA free-energy decomposition for the A296S mutant receptor bound to fipronil (red bars) and fluralaner (blue bars); Figure S2: Time-resolved MM/PBSA binding free energy for the A296S mutant receptor in complex with fipronil (red line) or fluralaner (blue line) across the last 100 ns production trajectories; Figure S3: Per-residue MM/PBSA free-energy decomposition for the wild-type receptor bound to fipronil (red bars) and fluralaner (blue bars); Figure S4: Time-resolved MM/PBSA binding free energy for the wild-type receptor in complex with fipronil (red line) or fluralaner (blue line) across the last 100 ns production trajectories; Figure S5: Grid box defining the search space for ligand docking within the M1–M3 intersubunit cavity of the pentamer; Table S1: Quality metrics of the structural models. Values for the Phyre2.2 monomers and the AlphaFold monomer refer to single subunits; Figure S6: AlphaFold monomer confidence and superposition with the Phyre2.2 monomer; Figure S7: Superpositions involving the monomer built on the honeybee RDL template (PDB 9SIO); Figure S8: Pentameric assemblies.

## Abbreviations

The following abbreviations are used in this manuscript:

COM	Center of mass
GABA	γ-Aminobutyric acid
GAFF2	General Amber Force Field, version 2
IE	Interaction entropy
IRAC	Insecticide Resistance Action Committee
LINCS	Linear Constraint Solver
MD	Molecular dynamics
MM/PBSA	Molecular Mechanics/Poisson–Boltzmann Surface Area
NCA	Non-competitive antagonist
NPT	Isothermal–isobaric ensemble (constant number of particles, pressure, and temperature)
NVT	Canonical ensemble (constant number of particles, volume, and temperature)
OPM	Orientation of Proteins in Membranes
pLGIC	Pentameric ligand-gated ion channel
PLIP	Protein–Ligand Interaction Profiler
POPC	1-Palmitoyl-2-oleoyl-glycero-3-phosphocholine
PPM	Positioning of Proteins in Membranes
RDL	Resistance to Dieldrin
RMSD	Root-mean-square deviation
RMSF	Root-mean-square fluctuation
RoG	Radius of gyration
SASA	Solvent-accessible surface area
TIP3P	Transferable Intermolecular Potential with 3 Points (water model)
WHO	World Health Organization
WT	Wild-type
