# Supplementary material for: Ligand-Specific Remodeling of the Anopheles gambiae RDL GABA Receptor by the A296S Mutation Differentiates Recognition of Fipronil and Fluralaner
Source: Biology (Basel). 2026 Nov 5;15(21):2011. doi: 10.3390/biology15212011 (PMC13761679; doi:10.3390/biology15212011)
Supplement: Supplementary file 1 [file biology-15-02011-s001.zip › biology-4523209-supplementary.pdf]

Supplementary Materials

The following figures and tables provide additional structural and per-residue detail supporting the main-text analyses.

Supplementary Table S1 and Figures S6–S8 report the structural-model comparison and quality assessment described in Section 2.1 of the main text.

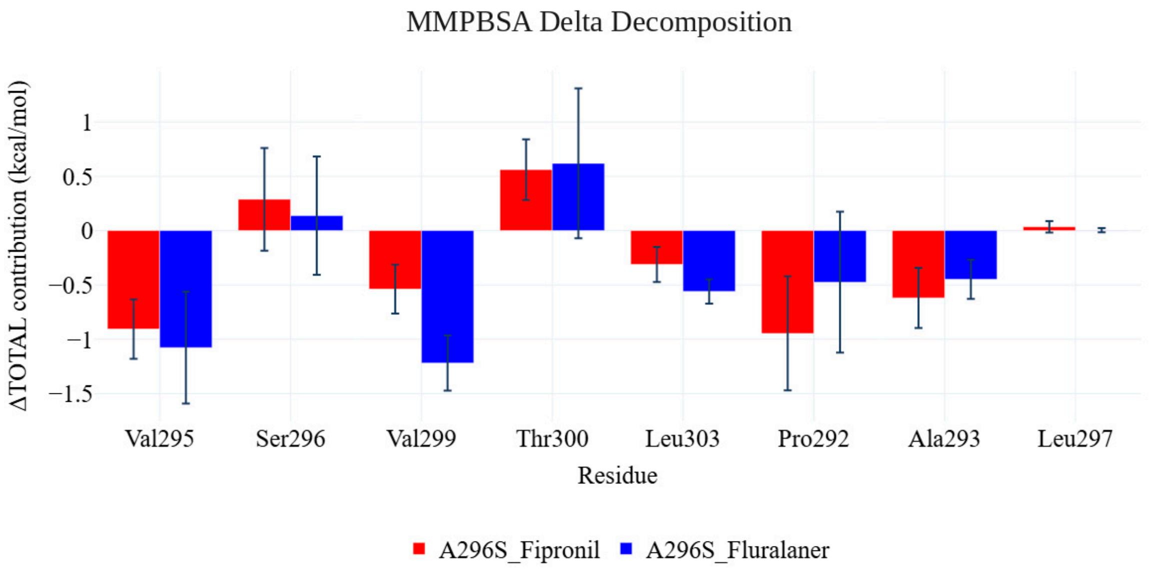

Figure S1. Per-residue MM/PBSA free-energy decomposition for the A296S mutant receptor bound to fipronil (red bars) and fluralaner (blue bars). Bars represent the binding free-energy contribution (kcal/mol) of individual amino acid residues located within 0.5 nm of the bound ligand, labeled by chain identity and residue name. The mean value over three replicates is shown with standard deviation as shades (mean ± s.d).

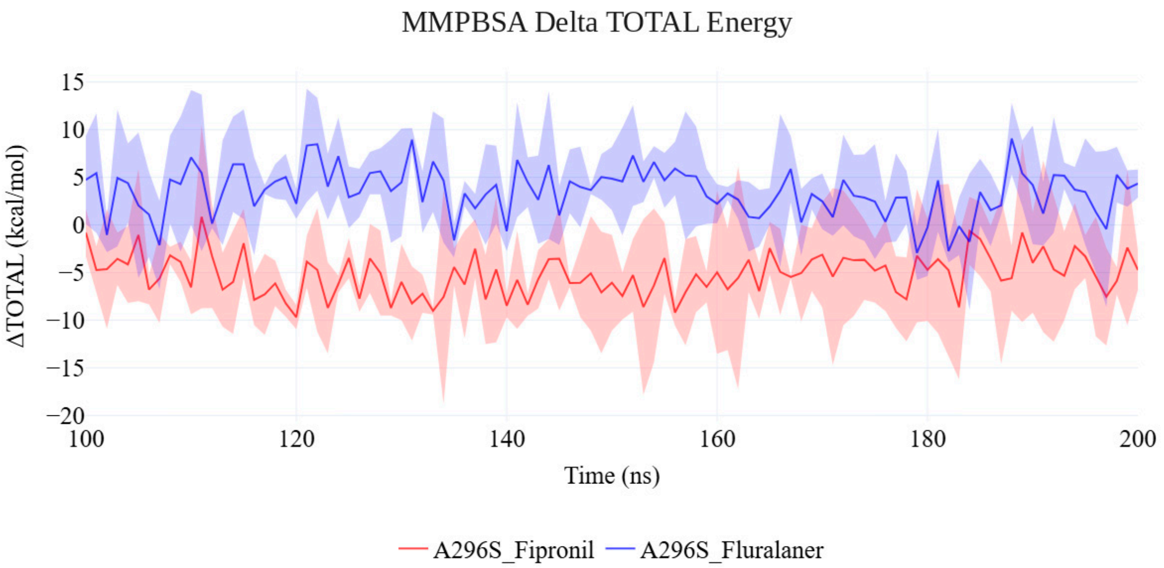

Figure S2. Time-resolved MM/PBSA binding free energy for the A296S mutant receptor in complex with fipronil (red line) or fluralaner (blue line) across the last 100 ns production trajectories. The mean value over three replicates is shown with standard deviation as shades (mean ± s.d).

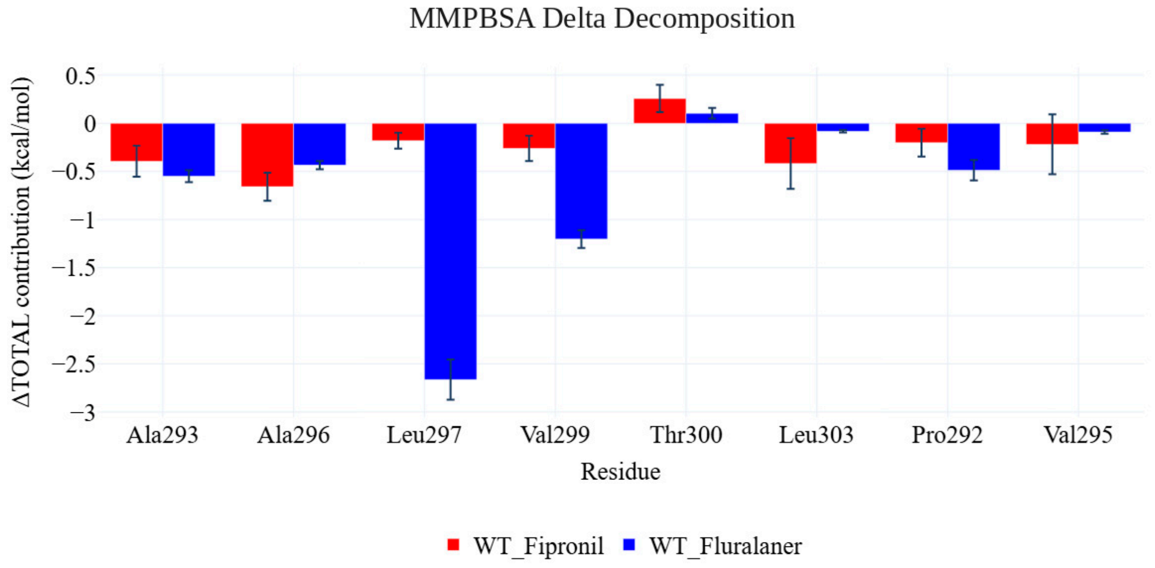

Figure S3. Per-residue MM/PBSA free-energy decomposition for the wild-type receptor bound to fipronil (red bars) and fluralaner (blue bars). Bars represent the binding free-energy contribution (kcal/mol) of individual amino acid residues located within 0.5 nm of the bound ligand, labeled by chain identity and residue name. The mean value over three replicates is shown with standard deviation as shades (mean ± s.d).

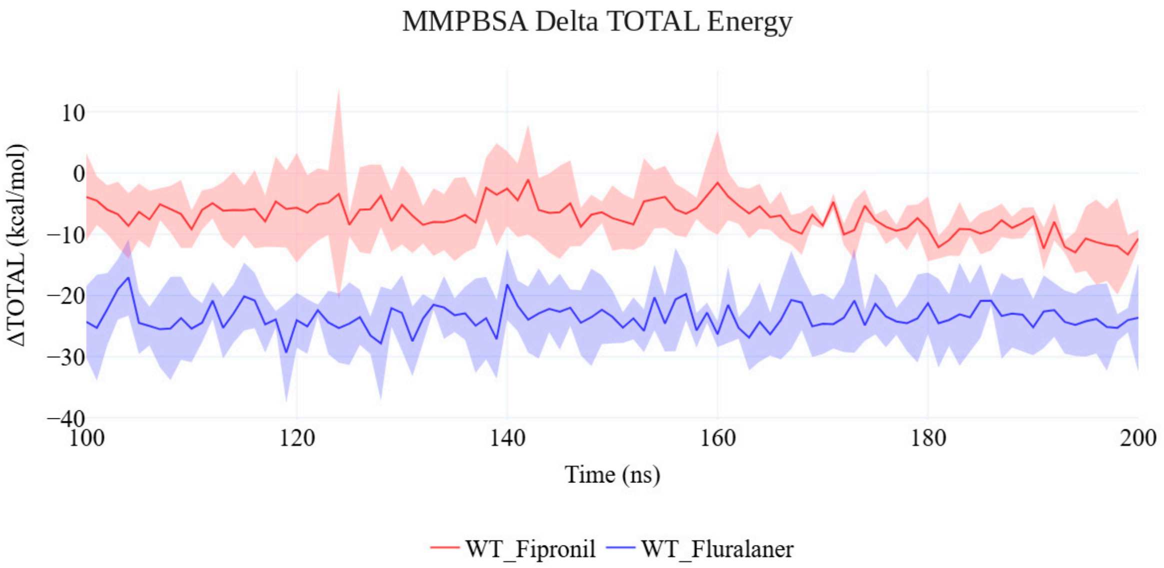

Figure S4. Time-resolved MM/PBSA binding free energy for the wild-type receptor in complex with fipronil (red line) or fluralaner (blue line) across the last 100 ns production trajectories. The mean value over three replicates is shown with standard deviation as shades (mean ± s.d).

a

b

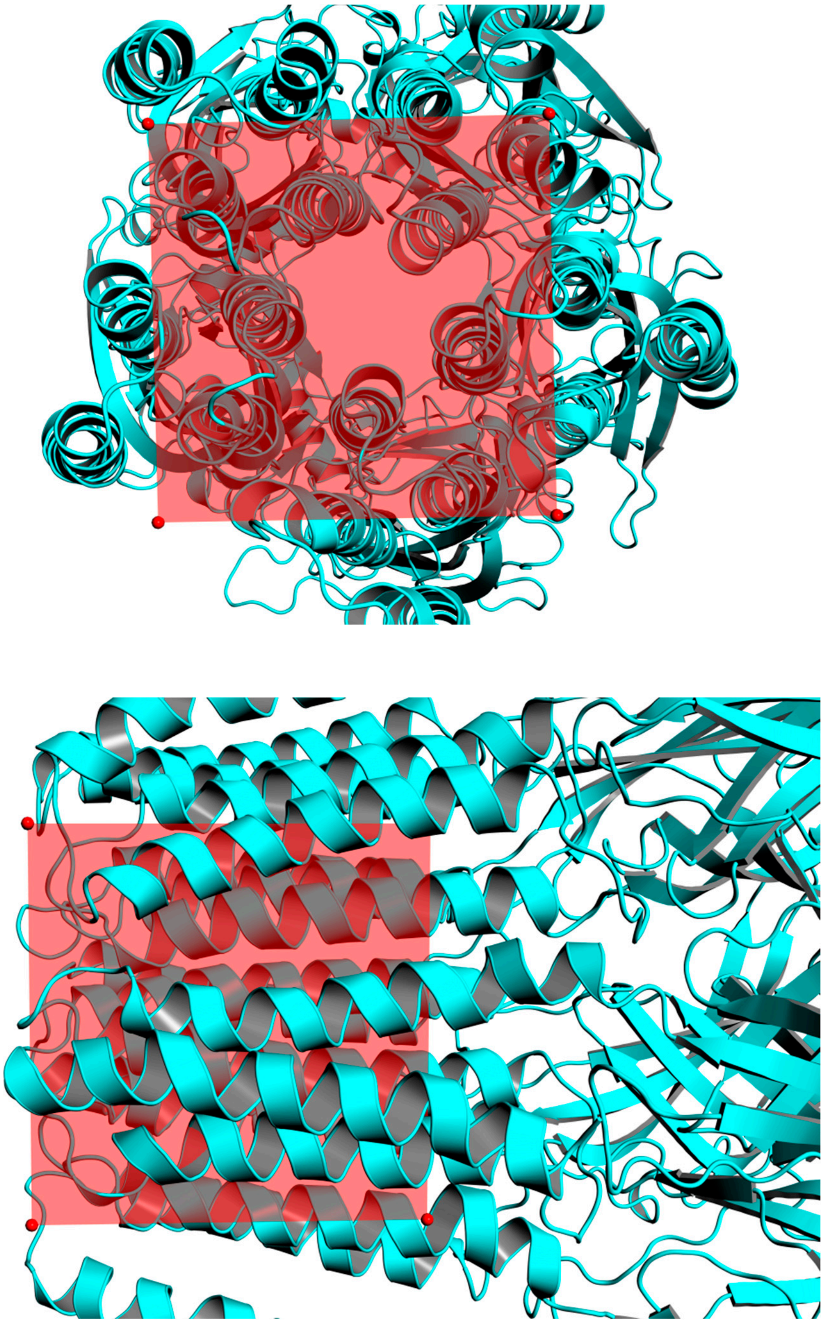

Figure S5. Grid box defining the search space for ligand docking within the M1–M3 intersubunit cavity of the pentamer. (a) Top view along the z-axis, showing the grid box covering the helices lining the pore lumen. (b) Side view.

Structural-model comparison and quality assessment*

The Phyre2.2 monomer used for the simulations (template PDB 7QN8, chain D; 48% sequence identity; 326 aligned residues) and the refined homopentamer were compared with two additional models: the AlphaFold2 model of full-length RDL retrieved from the AlphaFold Protein Structure Database (entry AF-A0A1I9VT68-F1) [23,24], and a second Phyre2.2 monomer built on the honeybee RDL cryo-EM structure (PDB 9SIO, an apo structure) [3]. PDB 7QN8 is a human β3δ GABA(A) receptor structure determined with histamine and nanobody Nb25 [19] and is not an insect RDL structure. Stereochemical and model-quality metrics were obtained with the structure assessment server [25] and are given in Table S1. Pairwise RMSD and TM-score values between monomers were calculated with TM-align [26] (normalized by model), and the refined homopentamer was compared with 9SIO using the structure assessment server [25] (Figures S6–S8).

The AlphaFold monomer contains extended low-confidence, disordered regions in the N-terminal (extracellular) and C-terminal (intracellular) segments (Figure S6a). Its Ramachandran outlier fraction (7.23%) is higher than those of the other models, so its stereochemical metrics are not uniformly better than those of the homology models. The Phyre2.2 monomer, the honeybee-template monomer, and the AlphaFold monomer superposed with RMSD values of 1.38–1.65 Å and TM-scores of 0.938–0.969, and the refined homopentamer superposed on the honeybee RDL structure with an RMSD of 2.24 Å and a TM-score of 0.94 (Figures S6–S8). These values indicate agreement of the overall fold.

Limitations. Most values are global measures, although the lDDT of the refined homopentamer against 9SIO (0.67) is a local-distance measure that adds some local information. No M2-specific RMSD and no local deviation at residue 296 were calculated. The PAE matrix is specific to the AlphaFold prediction, has no counterpart for the other models, and can be viewed on the database entry (AF-A0A1I9VT68-F1); it was not analyzed here. The comparison therefore cannot establish that the M1–M3 binding pocket is equivalent among the models, and it does not validate the docking poses or the MM/PBSA energies. The honeybee structure 9SIO is an apo structure, whereas 7QN8 was determined with histamine bound, so part of the pentamer deviation may reflect a difference in conformational state. A pentamer assembled from the AlphaFold monomer (Figure S8b) is disordered at the long terminal regions and is not independent validation of the assembly. The receptor model used here has not

been validated experimentally for *An*. *gambiae* RDL, and results depend on the conformational state of the template.

* References are numbered according to their order of appearance in the main manuscript.

Table S1. Quality metrics of the structural models. Values for the Phyre2.2 monomers and the AlphaFold monomer refer to single subunits; the last column refers to the refined homopentamer. "Not reported" indicates that the value was not obtained.

**Metric**	**Phyre2.2 monomer (7QN8, chain D)**	**AlphaFold monomer (full length)**	**Phyre2.2 monomer (honeybee RDL 9SIO template)**	**Refined homopentamer**
QMEAN4	-3.58	Not reported	-2.59	-2.18
QMEANDisCo	0.80 ± 0.05	Not reported	0.88 ± 0.05	0.78 ± 0.05
MolProbity score	2.22	1.93	2.17	1.57
Ramachandran favored (%)	97.24	84.99	97.62	98.47
Ramachandran outliers (%)	0.92	7.23	0.0	0.0
Mean pLDDT	Not applicable	73.19	Not applicable	Not applicable
PAE	Not applicable	Not analyzed (see database entry)	Not applicable	Not applicable

a b

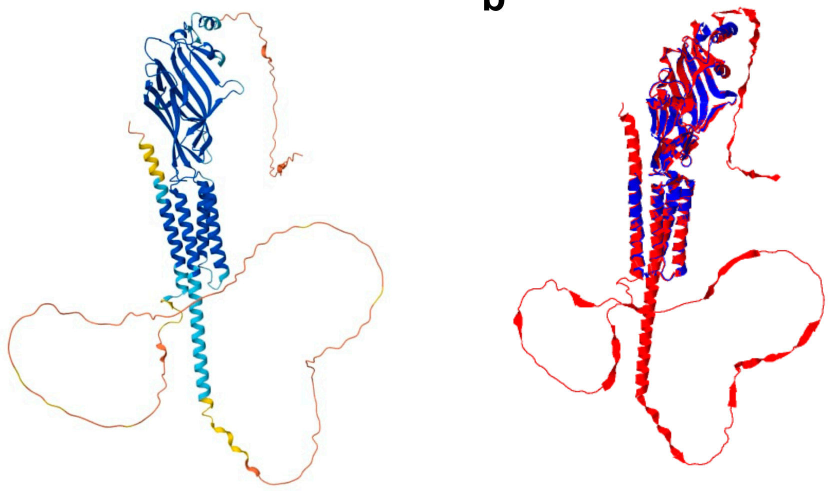

Figure S6. AlphaFold monomer confidence and superposition with the Phyre2.2 monomer. (a) AlphaFold2 model of full-length RDL (database entry AF-A0A1I9VT68-F1) colored by per-residue pLDDT (mean 73.19); the extended terminal segments are low-confidence and disordered. (b) Superposition of the Phyre2.2 monomer (blue) on the AlphaFold monomer (red): RMSD = 1.38 Å; TM-score = 0.96896 (normalized by model).

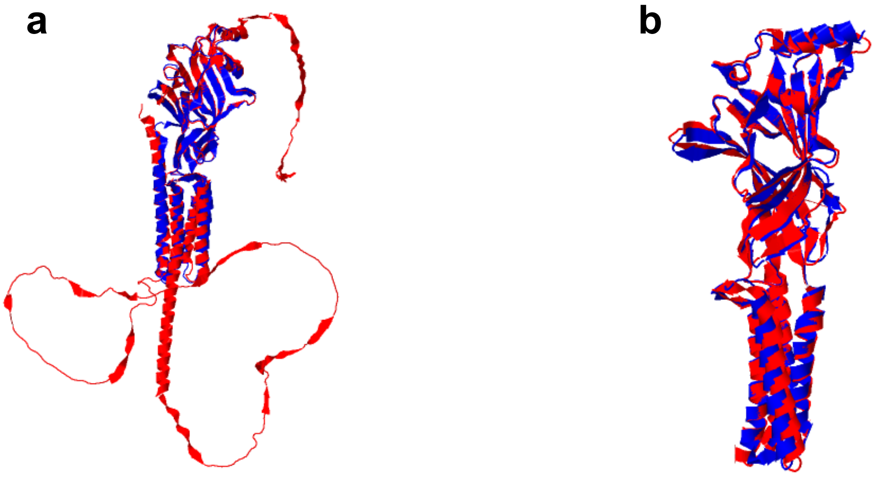

Figure S7. Superpositions involving the monomer built on the honeybee RDL template (PDB 9SIO). (a) Honeybee-template monomer (blue) on the AlphaFold monomer (red): RMSD = 1.56 Å; TM-score = 0.95353 (normalized by model). (b) Phyre2.2 monomer used in this study (blue) on the honeybee-template monomer (red): RMSD = 1.65 Å; TM-score = 0.93803 (normalized by model).

a b

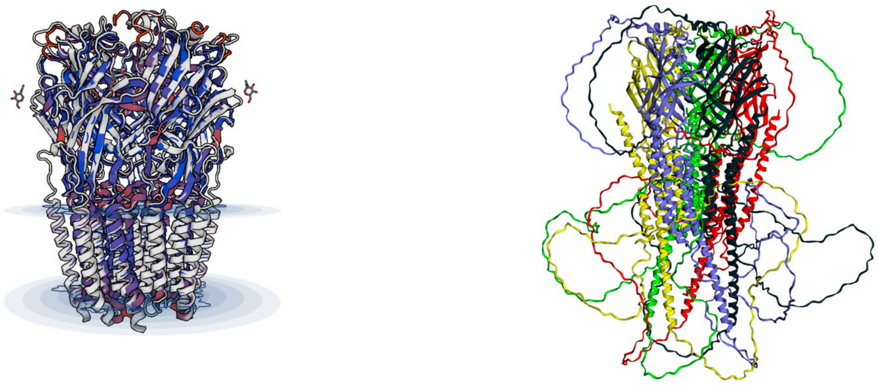

Figure S8. Pentameric assemblies. (a) Refined homopentamer (colored) superposed on the honeybee RDL cryo-EM structure 9SIO (white): lDDT = 0.67; RMSD = 2.24 Å; TM-score = 0.94; global QS-score = 0.71; global QS-best = 0.73; DockQ = 0.59. (b) Pentamer assembled from the AlphaFold monomer, showing extended disordered N- and C-terminal regions; this assembly is shown for comparison only and is not used as independent validation.

Supplementary References

[3] Labouré, T.; Pandey, M.P.; Zarkadas, E.; Juillan-Binard, C.; Baud, D.; Neyton, J.; Cens, T.; Rousset, M.; Dehez, F.; Charnet, P.; Nury, H. Structures of the honeybee $GABA_a$ RDL receptor illuminate allosteric modulation. *Neuron* **2026**, *114*, 1234–1245. https://doi.org/10.1016/j.neuron.2025.12.013

[19] Sente, A.; Desai, R.; Naydenova, K.; Malinauskas, T.; Jounaidi, Y.; Miehling, J.; Zhou, X.; Masiulis, S.; Hardwick, S.W.; Chirgadze, D.Y.; Miller, K.W.; Aricescu, A.R. Differential assembly diversifies GABAA receptor structures and signalling. Nature 2022, 604, 190–194. https://doi.org/10.1038/s41586-022-04517-3

[23] Jumper, J.; Evans, R.; Pritzel, A.; Green, T.; Figurnov, M.; Ronneberger, O.; Tunyasuvunakool, K.; Bates, R.; Žídek, A.; Potapenko, A.; Bridgland, A.; Meyer, C.; Kohl, S.A.A.; Ballard, A.J.; Cowie, A.; Romera-Paredes, B.; Nikolov, S.; Jain, R.; Adler, J.; et al. Highly accurate protein structure prediction with AlphaFold. Nature 2021, 596, 583–589. https://doi.org/10.1038/s41586-021-03819-2

[24] Varadi, M.; Bertoni, D.; Magana, P.; Paramval, U.; Pidruchna, I.; Radhakrishnan, M.; Tsenkov, M.; Nair, S.; Mirdita, M.; Yeo, J.; Kovalevskiy, O.; Tunyasuvunakool, K.; Laydon, A.; Žídek, A.; Tomlinson, H.; Hariharan, D.; Abrahamson, J.; Green, T.; Jumper, J.; et al. AlphaFold Protein Structure Database in 2024: Providing structure coverage for over 214 million protein sequences. Nucleic Acids Res. 2024, 52, D368–D375. https://doi.org/10.1093/nar/gkad1011

[25] Waterhouse, A.M.; Studer, G.; Robin, X.; Bienert, S.; Tauriello, G.; Schwede, T. The structure assessment web server: for proteins, complexes and more. Nucleic Acids Res. 2024, 52, W318–W323. https://doi.org/10.1093/nar/gkae270

[26] Zhang, Y.; Skolnick, J. TM-align: A protein structure alignment algorithm based on the TM-score. Nucleic Acids Res. 2005, 33, 2302–2309. https://doi.org/10.1093/nar/gki524
